# Experiences of maternity care among women at increased risk of preterm birth receiving midwifery continuity of care compared to women receiving standard care: Results from the POPPIE pilot trial

**DOI:** 10.1371/journal.pone.0248588

**Published:** 2021-04-21

**Authors:** Cristina Fernandez Turienzo, Sergio A. Silverio, Kirstie Coxon, Lia Brigante, Paul T. Seed, Andrew H. Shennan, Jane Sandall, On behalf of the POPPIE Collaborative Group

**Affiliations:** 1 Department of Women and Children’s Health, Faculty of Life Science and Medicine, King’s College London, London, United Kingdom; 2 Department of Midwifery, Faculty of Health, Social Care and Education, Kingston University and St. George’s, University of London, London, United Kingdom; 3 Department of Midwifery, Florence Nightingale Faculty of Nursing and Midwifery, King’s College London, London, United Kingdom; Ospedale dei Bambini Vittore Buzzi, ITALY

## Abstract

**Background:**

Midwifery continuity of care models for women at low and mixed risk of complications have been shown to improve women’s experiences of care. However, there is limited research on care experiences among women at increased risk of preterm birth. We aimed to explore the experiences of care among women with risk factors for preterm birth participating in a pilot trial (POPPIE) of a midwifery continuity of care model which included a specialist obstetric clinic.

**Methods:**

A total of 334 pregnant women identified at increased risk of preterm birth were randomly allocated to either midwifery continuity of care (POPPIE group) or standard maternity care. Women in both groups were followed up at six-to-eight weeks postpartum and were invited to complete a postnatal survey either online or by post. An equal status exploratory sequential mixed method design was chosen to collect and analyse the quantitative postnatal survey data and qualitative interviews data. The postnatal survey included measures of social support, trust, perceptions of safety, quality of care, control during childbirth, bonding and quality of life. Categorical data were analysed with chi-squared tests and continuous data were analysed with t-tests and/or Mann-Whitney U test to measure differences in measures scores among groups. The qualitative interview data were subjected to a thematic framework analysis. Data triangulation brought quantitative and qualitative data together at the interpretation stage.

**Findings:**

A total of 166 women completed the survey and 30 women were interviewed (90 and 16 in POPPIE group; 76 and 14 in standard group). We found survey respondents in the POPPIE group, compared to respondents in the standard group, were significantly more likely to report greater trust in midwives (Mann-Whitney U, p<0.0001), greater perceptions of safety during the antenatal care (t-test, p = 0.0138), have a particular midwife to contact when they needed during their pregnancy (t-test, p<0.0001) and the postnatal period (chi-squared, p<0.0001). They reported increased involvement in decisions regarding antenatal, intrapartum and postnatal care (t-test, p = 0.002; p = 0.008; p = 0.006 respectively); and greater postnatal support and advice about: feeding the baby (chi-squared, p<0.0001), handling, settling and looking after the baby (chi-squared, p<0.0001), baby’s health and progress (chi-squared, p = 0.039), their own health and recovery (chi-squared, p = 0.006) and who to contact about any emotional changes (chi-squared, p = 0.005). There were no significant differences between groups in the reporting of perceptions of safety during birth and the postnatal period, concerns raised during labour and birth taken seriously, being left alone during childbirth at a time of worries, control during labour, bonding, social support, and physical and mental health related quality of life after birth. Results from qualitative interviews provided insight and depth into many of these findings, with women in the POPPIE group reporting more positive experiences of bonding towards their babies and more positive physical health postnatally.

**Conclusions:**

Compared with standard maternity care, women at increased risk of PTB who received midwifery continuity of care were more likely to report increased perceptions of trust, safety and quality of care.

**Trial registration:**

ISRCTN (Number: 37733900); UK CRN (ID: 31951).

## Introduction

Preterm birth (PTB) is the term used to define any birth before 37 weeks of completed gestation. One in ten babies worldwide are born too soon and over a million die from related complications [1]. Many preterm babies do survive but they are specifically vulnerable to significant health conditions and disabilities which impact on families, societies and health systems [2]. Despite efforts to decrease the prevalence, improve clinical management and reduce neonatal mortality and morbidity, PTB continue to rise in most countries [3]. Most PTBs are spontaneous and only a small proportion are provider-initiated due to maternal or maternal reasons, yet the cause is still unknown in up to half of the cases and involves multiple and overlapping factors (e.g. chronic diseases, infections, poor obstetric history such as previous PTB or late miscarriages, psychosocial stress, smoking, domestic violence) [4]. Thus, achievement of public health strategies to prevent PTB has been challenging.

A Cochrane review of reviews found that midwifery continuity of care models for pregnant women at low and mixed risk of complications are the only health service and system interventions shown to have both a reduction in PTB and improvement in perinatal survival [5]. Women who received care by a named midwife or a small group of midwives during pregnancy, birth, and the postnatal periods were 24% less likely to have a PTB and more likely to have better maternal and infant outcomes and report more satisfaction with care [6]. Most included trials reported overall greater satisfaction with various aspects of care in continuity models (e.g. explanations, information, choices, preparation for labour, control, behaviour of staff); however, most trials did not include women with high risk pregnancies, used a wide variety of instruments, scales and outcomes, and only a few focused on the experiences of childbirth [6]. No trial evaluating a continuity model for at risk women has explicitly reported other important outcome measures such as access to care, trust, quality and safety, care or coordination and navigation through healthcare systems; these domains are thought to be particularly relevant for high and mixed risk populations.

A recent study exploring women’s experiences of risk and care management found that clinicians should promote continuity of care models for women attending high risk clinics such as those specialised in preterm surveillance [7]. In addition, the Saving Lives Care Bundle, a group of actions that have been put together to reduce stillbirth in the UK, also recommends continuity of care should be followed in conjunction with the care bundle [8]. However, the effect of midwifery continuity of care on the experiences of women who are at high risk of PTB remain unknown and more research is needed to understand mechanisms by which continuity models may reduce PTB. Continuity models are at the heart of maternal policy in the United Kingdom and recommended in Australia [9, 10], and the latest WHO antenatal and intrapartum care guidelines for a positive pregnancy and childbirth experience, recommend these models for pregnant women in settings with well-functioning midwifery programmes [11, 12]. However, understanding what the important core elements are for women, and what can be adapted to context to achieve the beneficial outcomes for different risk populations, is crucial for implementing and scaling up sustainable models of midwifery continuity of care [13].

The results of the first pilot randomised controlled trial to evaluate a model of midwifery continuity of care linked with a specialist obstetric clinic for women at increased risk for PTB were recently published [14]. When assessing feasibility, fidelity and clinical outcomes, authors found that it was feasible to set up and maintain fidelity to the model indicating that a full scale RCT is possible, but the model did not improve the clinical composite outcome of appropriate and timely interventions for the prevention and/or management of preterm labour and birth, in this setting, for this very high-risk population group. Thus, hypothesised mechanisms such as to how the model might have worked (increased trust and engagement, improved care coordination and earlier referral) might have limited influence where there were pathological physiological mechanisms such as in PTB. This paper aims to assess the effect on maternal experiences of maternity care among women at risk of PTB who received midwifery continuity of care (POPPIE group) and women who received standard care (standard group). The two main objectives are: 1) to quantitatively measure and compare perceptions of social support, trust, safety and quality, control during childbirth, bonding and quality of life among women in both groups; and 2) to explore those concepts using qualitative methods to get a deeper understanding of specific experiences and potential mechanisms.

## Methods

### Study design and participants

We employed an equal status exploratory sequential mixed method design with a methodology grounded in pragmatism [15, 16]. A cross-sectional postnatal survey followed by qualitative interviews were used with women participating in the POPPIE trial. This approach was specifically chosen in order to collect and analyse quantitative survey data and collect and analyse qualitative interview data to add deeper understanding of experiences and potential mechanisms to initial quantitative results. Both the quantitative and qualitative components were equally valued to answer our research questions [17]. The GRAMMS reporting guidelines for mixed methods research were followed [18].

The main POPPIE trial paper is described in detail elsewhere [19], but in brief, this study used a two-arm hybrid implementation-effectiveness, randomised, controlled pilot trial within an inner-city teaching hospital in the UK between, to compare midwifery continuity of care (POPPIE group) with standard maternity care (standard group) for women identified at increased risk of PTB. Pregnant women attending for antenatal care at less than 24 weeks’ gestation were eligible if they were considered at risk of preterm birth (e.g. previous cervical surgery, preterm birth, late miscarriage; smokers). Women aged less than 18 years and those with multiple pregnancy or already receiving care from a specialist midwifery team (e.g. severe mental illness, substance misuse) were excluded. Pregnant women were recruited to the study by research assistants and midwives at their antenatal or ultrasound scan appointment and randomly assigned in a 1:1 ratio. Women allocated to the POPPIE group received antenatal, intrapartum and postnatal care in the hospital, community or at home, predominantly from a named (or primary) midwife, who worked with a partner midwife within a small team, known as the POPPIE team; women allocated to the standard group received standard maternity care provided by different midwives working in the community, children’s centres and/or hospital. In accordance with the hospital guidelines, women in both POPPIE and standard groups followed the same obstetric care pathway.

Women in the POPPIE and standard groups who had a livebirth and did not withdraw from the trial were sent greeting cards after birth and were followed up at six-eight weeks postpartum (or until discharge from neonatal intensive care unit up to three months) to be invited to complete a postnatal survey either online or by post. Up to three reminder phone calls and/or texts were sent every two weeks to non-responders. Women in both groups were also invited to take part in qualitative interviews based on a maximum variation sampling strategy taking into account key factors related to PTB such as socio-demographic characteristics and obstetric history.

Regulatory and ethical approvals were obtained (London South East Research Ethics Committee; Ref 17/LO/0029; ID 214196) and written consent was provided by all women participating in the trial and interviews. The pilot was overseen throughout by a trial management group and an independent trial steering committee with representation from an obstetrician, a neonatologist, two senior midwives and a lay advisor contributing to patient and public involvement and engagement (PPIE).

### Outcome measure

The feasibility outcomes of the pilot trial included eligibility, recruitment and attrition rates, and fidelity of the model. The primary clinical outcome was a composite of timely and appropriate interventions for the prevention and/or management of preterm labour and birth. This paper presents secondary outcomes on women’s views and experiences of maternity care.

### Data collection

Baseline demographic characteristics were collected through self-administered questionnaires completed at recruitment. Clinical and outcome data for mother and babies were abstracted from medical records and electronic data systems, and experiential outcomes collected from 1) the postnatal survey and 2) qualitative interviews.

#### 1) Postnatal survey

The postnatal survey was partially based on questionnaires from previous studies of models of care and maternity surveys conducted in the UK [20, 21] and included standardised psychometric scales to measure different aspects of women’s experiences during pregnancy, birth and postpartum. It was piloted with the PPI group (which included parents of preterm babies) whose feedback helped to amend and re-frame important measures. The following standardised scales and questions were used:

### Social Support Scale (SSS) [22]

The 10-item SSS was specifically devised for pregnant and postpartum women in the Avon Longitudinal Study of Pregnancy and Childhood (ALSPAC) study to measure social support in relation to emotional, instrumental, and financial aspects. Participants respond using one of four categories from *‘I never feel this way’* through to *‘this is exactly how I feel’*. Possible total scores for the SSS range from 4 (indicating better social support) to 40 (reflecting worse social support). The SSS used in this study has demonstrated internal consistency with a Cronbach’s alpha of 0.77 (S1 File).

#### Trust in Nurses Scale (TNS) [23]

The 5-item TNS adapted for midwives was used to measure trust in midwives as an outcome of maternity care processes. Each item addresses a midwife activity or patient feeling. Responses are on a Likert scale (1 = *‘Never’* to 6 = *‘Always’*). Possible total scores range from 5 (indicating low trust) to 30 (indicating high trust). An additional global item question of the scale asks participants to write a number between 1 and 10 to rate her trust on midwives (higher score reflects higher trust). The adapted TNS version for midwives had good construct validity and high internal consistency reliability (Cronbach’s alpha = 0.93) (S2 File).

### Perceptions of safety scale [24]

The 13-item scale in the Perceptions of Safety Measurement Questionnaire [24] was shortened and adapted to measure perceptions of safety within a maternity hospital setting. Seven items were used to measure safety in relation to support, communication and consent, clinical interventions, staff workforce, familiarly with equipment and procedures, medication information and discharge process. Responses are on a Likert scale (1 = ‘less important in making you feel safe’ to 5 = ‘most important in making you feel safe’). Possible total scores range from 5 (indicating less important in feeling safe) to 35 (indicating most important). Validity and internal consistency of the 7-item scale was good (Cronbach’s alpha = 0.80) (S3 File).

Additional safety and quality related questions included: whether women were able to contact the midwife when needed during pregnancy and after birth (Yes/No), and if yes how; whether women were spoken in a way they could understand (Yes/No); whether they would have preferred to have been more or less involved in the decisions about their maternity care (Less involved/More involved/Happy with how involved); whether raised a concern during labour and birth felt that they was taken seriously (Yes/No); whether women (and companions if women had one) were left alone during labour and birth at a worrying time (Yes/No); and whether specific advice and help during the postnatal period was provided (e.g. feeding the baby, own health and recovery) (Yes/No). Details of additional questions and scales items can be found in S4 File.

### The Labour Agentry Scale (LAS) [25]

The shortened version of the LAS consists of 10 affirmative statements to measure control during childbirth (e.g. ‘I felt confident’ and ‘I felt tense’). Women’s degree of agreement or disagreement with each item is measured on a 7-point Likert scale from 1 (rarely) to 7 (almost always). Possible total scores for the LAS range from 10 (rarely felt in control) to 70 (almost always felt in control). Negatively worded items such as “tense” or “hopeless” are reversed so that a higher score reflects a better control. The internal consistency of the LAS ranged from 0.91 to 0.98 (with LAS scores remaining stable at 2 weeks, 1 month and 3 months postpartum) with a Cronbach’s alpha of 0.93 [25].

### Mother-Infant Bonding Scale (MIBS) [26]

The 8-item self-rating MIBS assesses the maternal feelings for the child in the first few weeks and includes items such as “disappointed” or “resentful” on a four-point Likert scale (where 0 = “not at all” and 3 = “very much”). Thus, a high score indicates worse mother to infant bonding. Positively worded items such as “loving” or “joyful” are reversed so that a lower score reflects a better bonding. Possible total scores for the MBIS range from 0 (indicating better bonding) to 24 (indicating worse bonding). Reliability analysis demonstrated a Cronbach’s score of 0.71 [26].

### Patient reported outcomes measurement information system global health (PROMIS-10 global) [27]

The PROMIS-10 Global measures physical and mental health domains including overall physical health, mental health, social health, pain, fatigue, and overall perceived quality of life [27]. Response options are presented as 5-point rating scales (with a single additional 11-point scale). The results of the questions are used to calculate two summary scores: a Global Physical Health (GPH) Score and a Global Mental Health (GMH) score. These scores are then standardised with a mean (SD) of 50 (10) for the US general population where higher scores indicate better outcome. The cut-off points or thresholds for PROMIS Global Physical and Mental T scores were developed based on categories of excellent, very good, good, fair, and poor where respective cut points are, for GMH: 56, 48, 40, 29, and for GPH: 58, 50, 42, 35. The GPH and GMH scales have internal consistency reliability coefficients of 0.81 and 0.86 respectively [27]. A comparison of PROMIS-10 Global and the EuroQol five-dimension (EQ-5D) questionnaires suggested either is appropriate to evaluate health-related quality-of-life outcomes among some patients in clinical studies in the UK [28].

#### 2) Qualitative interviews

Semi-structured qualitative interviews (n = 30) were used as this interview style allows for important questions to be answered by participants and provides the flexibility for the interviewer to follow-up on points made by participants which are pertinent to their experiences [29]. The interview topic guide is presented in S5 File. Women were asked to share their experiences in their journey through antenatal, intrapartum and postnatal care. Interviews were conducted based on participants’ preferences (most face to face at their home and few over the phone) by two researchers with postgraduate training in qualitative research. They were nurses and/or midwives by background themselves and completed reflective diaries after each interview to encourage ongoing reflexivivity [30]. Interviews lasted one hour on average and were digitally recorded and transcribed verbatim, and then uploaded and managed in NVivo software (version 12).

### Quantitative and qualitative analysis

The postnatal survey included categorical data which were analysed with *χ*^2^ tests and continuous data which were analysed with *t* tests (for normally distributed data) and Mann-Whitney U test for non-normally distributed data to measure differences in measures scores among women in the POPPIE and standard groups using STATA software (version 15).

Qualitative interview data were subjected to a thematic framework analysis, which has seven data processing stages: transcription, familiarization, coding, developing an analytical framework, applying the framework, charting data into a framework matrix and interpreting data [31]. The scales and additional questions used in the survey were used to build the overarching analytical framework. Key parts of each scale and questions provided the coding structure which was set up in NVivo where interview transcripts were analysed. Coding followed this framework matrix, and once complete, data were stratified by participant characteristics (trial group, ethnicity, risk factors) to allow for differential interpretation, whereby the most relevant quotations were then reported in the text. To ensure rigour, transcripts were coded by two researchers and inter-analyst reliability was deemed high [32].

### Mixed-methods triangulation

Data triangulation followed a parallel approach [16], whereby quantitative and qualitative data collection and analysis were undertaken separately and only brought together at the interpretation stage [33]. This is a pragmatic approach to integration for large datasets and allowed for qualitative data to add depth to findings or questions which arose from quantitative data analysis [34]. Analysis and interpretation of these integrated data was therefore exploratory, reflecting guidance for mixed methods pilot trials [35].

## Results

Between 9 May 2017 and 30 September 2018, 334 women were recruited to the main pilot trial; 169 women were allocated to the POPPIE group and 165 to the standard group. Of the 149 women followed-up in the POPPIE group, 90 completed the postnatal survey; and out of the 154 women followed-up in the standard group, 76 completed the postnatal survey (response rate of 60.4% and 49.4% respectively). The overall survey response rate in both groups was 55%. A total of 20 women in the POPPIE group and 29 women in the standard group were invited for an interview and 16 and 14 accepted (acceptance rate of 80% and 48.3%, respectively). The overall interview acceptance rate was 64%.

Maternal characteristics at baseline are presented in Table 1. Overall, participants in the postnatal survey and interviews were similar between groups. Characteristics among survey respondents were similar to the overall POPPIE pilot trial sample: 29% ethnic minority groups, 61.2% living in most deprived areas, 58.9% with a university degree, 81% in employment, nearly 52% married with a total household weekly income of ≥ £650, 42.1% primiparous, 25% with at least one pre-existing medical condition, 22.4% with multiple obstetric risk factors for preterm birth, and 33.8% had at least one social risk factor.

**Table 1:** Maternal characteristics at baseline.

	Postnatal survey		Qualitative interviews	
Characteristic	POPPIE group (n = 90)	Standard group (n = 76)	POPPIE group (n = 16)	Standard group (n = 14)
Mean maternal age (years)	32.14 (5.39)	32.72 (5.29)	33.43 (1.30)	36.42 (1.21)
Ethnicity				
White	54 (60.0)	59 (77.6)	13 (81.2)	11 (78.6)
Black	16 (17.8)	8 (10.5)	2 (12.5)	3 (21.4)
Asian	10 (11.1)	2 (2.6)	1 (6.2)	0 (0.0)
Mixed	5 (5.6)	3 (3.9)	0 (0.0)	0 (0.0)
Other	5 (5.6)	4 (5.3)	0 (0.0)	0 (0.0)
Not fluent in English	2 (2.2)	0 (0.0)	0 (0.0)	1 (7.1)
Highest Educational level				
None	3 (3.3)	1 (1.3)	2 (12.5)	0 (0.0)
General Certificate of Education (or equivalent)	13 (14.4)	12 (15.8)	0 (0.0)	3 (21.4)
Vocational qualification	14 (15.6)	4 (5.3)	2 (12.5)	0 (0.0)
A level (or equivalent)	12 (13.3)	9 (11.8)	2 (12.5)	2 (14.3)
First degree/ Higher degree	47 (52.2)	50 (65.7)	12 (75.0)	9 (64.3)
Deprivation Index quintiles 1–2 (most deprived 40% of population) *	57/85 (63.3)	45/74 (59.2)	9 (56.2)	9 (64.3)
Current job situation				
Going to school / college full time	2 (2.2)	2 (2.6)	0 (0.0)	0 (0.0)
In paid employment/self-employed	70 (77.8)	64 (84.2)	13 (81.2)	13 (92.7)
Not doing paid work	7 (7.8)	6 (7.9)	2 (12.5)	1 (7.1)
Looking after home or family	11 (12.2)	2 (2.6)	1 (6.2)	0 (0.0)
Doing something else	0 (0.0)	2 (2.6)	0 (0.0)	0 (0.0)
Household income (gross/week)				
< £250	16 (17.8)	4 (5.3)	1 (6.2)	0 (0.00
£250-£350	7 (7.8)	3 (3.9)	1 (6.2)	1 (7.1)
£350-£450	5 (5.6)	6 (7.9)	1 (6.2)	0 (0.0)
£450-£650	8 (8.9)	3 (3.9)	1 (6.2)	3 (21.4)
>£650	39 (43.3)	45 (59.2)	10 (62.5)	8 (57.1)
Declined to answer	15 (16.7)	15 (19.7)	2 (12.5)	2 (12.5)
Marital or partner status				
Single (never married)	6 (6.7)	2 (2.6)	1 (6.2)	0 (0.0)
Married (and living with husband/wife)	42 (46.7)	45 (59.2)	12 (75.0)	12 (85.7)
Living with partner but not married (cohabitee)	23 (25.6)	23 (30.3)	2 (12.5)	2 (12.5)
Separated or divorced	2 (2.2)	1 (1.3)	0 (0.0)	0 (0.0)
In a relationship but not living together	5 (5.6)	4 (5.3)	0 (0.0)	0 (0.0)
Declined to answer	12 (13.3)	1 (1.3)	1 (6.25)	0 (0.0)
Mean gestation at booking (weeks)	9.91 (3.10)	9.95 (2.14)	9.00 (0.25)	8.92 (0.53)
Parity: nulliparous	32 (35.6)	37 (48.7)	7 (43.7)	6 (42.9)
Mean BMI (kg/m2)	26.19 (6.00)	24.67 (4.49)	24.68 (1.61)	24.26 (0.95)
Identified medical, obstetric, social risk				
*Pre-existing medical conditions*:				
Hypertension	2 (2.2)	1 (1.3)	1 (6.2)	0 (0.0)
Asthma	9 (10.0)	7 (9.2)	1 (6.2)	1 (7.1)
Autoimmune disease	3 (3.3)	2 (2.6)	1 (6.2)	0 (0.0)
Chronic renal disease	0 (0.0)	0 (0.0)	0 (0.0)	0 (0.0)
Chronic viral infection	1 (1.1)	0 (0.0)	0 (0.0)	0 (0.0)
Depression	11 (12.2)	8 (10.5)	1 (6.2)	2 (12.5)
Other mental health disorders	5 (5.6)	2 (2.6)	0 (0.0)	0 (0.0)
One pre-existing medical condition	17 (18.9)	18 (23.7)	2 (12.5)	3 (21.4)
Two or more pre-exiting medical conditions	6 (6.7)	1 (1.3)	2 (12.5)	1 (7.1)
Obstetric risk factors for PTB				
One or more PTBs (<37 weeks)	32 (35.6)	21 (27.6)	1 (6.2)	3 (21.4)
Previous cervical surgery (LLETZ, cone biopsy)	34 (37.8)	34 (44.7)	10 (62.5)	4 (62.5)
Previous PPROM (< 37 weeks)	14 (15.6)	8 (10.5)	0 (0.0)	0 (0.0)
Previous short cervix (<25mm)	7 (7.8)	3 (3.9)	0 (0.0)	0 (0.0)
Short cervix this pregnancy	2 (2.2)	2 (2.6)	0 (0.0)	1 (7.1)
Previous/ current failed cerclage	0 (0.0)	0 (0.0)	0 (0.0)	0 (0.0)
Uterine abnormality	3 (3.3)	1 (1.3)	0 (0.0)	0 (0.0)
Previous late loss (<24 weeks)	17 (18.9)	7 (9.2)	2 (12.5)	1 (7.1)
One obstetric risk factor	53 (58.9)	48 (63.2)	10 (62.5)	8 (57.1)
Two obstetric risk factors	19 (21.1)	10 (13.2)	2 (6.2)	4 (28.6)
Three or more obstetric risk factors	6 (6.7)	3 (3.9)	0 (0.0)	1 (7.1)
Social risk				
Smoker at booking	18 (20.0)	18 (23.7)	4 (26.7)	1 (7.1)
Past or present history of domestic violence	9 (10.5)	1 (1.3)	0 (0.0)	0 (0.0)
Past or present history of recreational drug use	8 (4.8)	12 (7.3)	0 (0.0)	0 (0.0)

Data are n (%) or mean (standard deviation). n/N (%) indicates that the denominator only includes participants with a relevant measurement for that variable PTB: preterm birth; PPROM: preterm premature rupture of membranes; LLETZ: large loop excision of the transformation zone.

* The Index of Multiple Deprivation is the method used to measure social and economic deprivation in small areas of England and Wales; a score of 1 is the highest and 5 the lowest. 7 postcodes were missing for women who completed the postnatal survey and could not be matched to database (Department for Communities and Local Government, 30/9/19; The English Indices of Deprivation 2019 statistical release).

Women who participated in interviews were similar in terms of primiparity (43.3%), social deprivation (60.2%) and medical and obstetric risk factors (26% and 20% respectively); however, less women were from ethnic minority groups (20%), more had a university degree (69.6%), were married (80.3%) with a household income of ≥ £650/pw (58%) and fewer had social risk factors (17.5%). We present findings below through the integration of the quantitative data from the survey scales and qualitative data from the interviews including illustrative quotations. We follow the woman’s chronological pathway in pregnancy through to the postnatal period. The main quantitative findings are also summarised In Table 2.

**Table 2:** Overview of survey quantitative findings.

Measure	POPPIE group (n = 90)	Standard group (n = 76)	Statistical test
			p value
Social Support Scale [22]	87/90	18.07 (4.62)	0.15
	17.82 (5.91)		(-1.35, 1.85)
			t-test; p = 0.7576
Trust in Nurses Scale [23] Adapted for Midwives†	87/90	24.68 (5.68)	-4.21
	28.89 (2.01)		(-5.44, -2.97)
			MWU; p<0.0001
Global Item: Rate your trust on a scale 0–10	87/90	8.14 (1.86)	-1.40
			(-1.83, -0.98)
	9.55 (0.79)		MWU; p<0.0001
Perceptions of Safety Scale [24] (AN)	25.31 (4.96)	23.28 (5.65)	-2.01
			(-3.01, 0.47)
			t-test; p = 0.0138
Perceptions of Safety Scale [24] (IP)	25.37 (5.49)	24.46 (5.87)	-0.91
			(-2.62, 0.78)
			t-test; p = 0.2902
Labour Agentry Scale [25]	52.12 (13.09)	50.69 (13.13)	-1.42
			(-5.37 to 2.52)
			t-test; p = 0.4775
Perceptions of Safety Scale [24] (PN)	87/90	27.78 (2.61)	-0.51
	28.28 (7.72)		(-2.69, 1.67)
			t-test; p = 0.6436
Additional safety/quality questions:			
Able to contact midwife when needed (AN)	89/90 (98.9)	51 (67.1)	χ^2^ test; p<0.0001
Able to contact midwife when needed (PN)	88/(100.0)	47 (61.8)	χ^2^ test; p<0.0001
Spoken in a way they could understand	90 (100.0)	74 (97.4)	χ^2^ test; p<0.0001
Felt happy with involvement in decisions (AN)	85 (94.4)	57 (75.0)	
Preferred to have been more involved in decision making (AN)	5 (5.5)	18 (23.6)	χ^2^ test; p = 0.002
Felt happy with involvement in decisions (IP)	78 (86.7)	51 (67.1)	
Preferred to have been more involved in decisions (IP)	12 (13.3)	51 (67.1)	χ^2^ test; p = 0.008
Felt happy with involvement in decisions (PN)	80/87 (91.9)	56 (73.68)	
Preferred to have been more involved in decisions (PN)	7/87 (8.05)	19 (25.0)	χ^2^ test; p = 0.006
Raised a concern during labour/birth and felt it was taken seriously	67 (74.4)	45 (59.2)	χ^2^ test; p = 0.063
Not left alone in labour/birth at a worrying time	69 (76.7)	53 (69.7)	χ^2^ test; p = 0.302
Always received specific advice and help during the PN period:			
Feeding the baby	71/88 (80.7)	37 (48.7)	χ^2^ test; p<0.0001
Handling, settling, looking after the baby	57/88 (64.7)	27 (35.5)	χ^2^ test; p<0.0001
Baby’s health, progress and any problems	69/88 (78.4)	46 (60.5)	χ^2^ test; p = 0.039
Own health and recovery after the birth	69/88 (78.4)	42 (55.3)	χ^2^ test; p = 0.006
Contact details for advice about emotional changes	64/88 (72.7)	36 (47.3)	χ^2^ test; p = 0.005
Mother-to-Infant Bonding Scale [26]	87/90	77/78	0.45
	1.54 (2.27)	1.97 (2.63)	(-0.28, 1.20)
			MMU; p = 0.2085
PROMIS-10 [27]: Health related quality of life (PN)			
Global Physical Health (GPH)	87/90	15.80 (2.29)	0.36
	15.43 (2.73)		(-0.42, 1.15)
T-score	47.7 (4.4)	47.7 (4.4)	t-test; p = 0.3610
Global Mental Health (GMH)	87/90	14.76 (3.37)	0.11
	14.65 (3.67)		(-0.98, 1.20)
T-score	48.3 (3.7)	48.3 (3.7)	t-test; p = 0.8461

Data are n (%), mean (standard deviation) or mean difference (95% confidence interval). n/N (%) indicates that the denominator only includes participants with a relevant measurement for that variable. MWU: Mann–Whitney U test; AN: Antenatal; IP: Intrapartum; PN: Postnatal.

### Social support

The SSS [21] scores reported in the postnatal survey by women in both groups ranged from 17.8 to 18.1, with a mean score of 18.3 indicating average/moderate levels of social support. There were no significant differences in mean SSS scores between women allocated to the POPPIE group and women allocated to standard group (0.15, -1.35 to 1.85; t-test, p = 0.7576).

More than a third of women interviewed in both groups reported social support mainly from partner, family and friends and peers, and a few reported support from neighbours.

*“It is horrible for a mother who has just given birth and you are already emotionally in a very bad mood and you cannot even hold your baby*, *and other mothers surrounded by their crying babies… obviously*, *my husband was there*. *If he was not there supporting me*, *I would have been in a very deep depression…”* (IW_30, POPPIE, White, one obstetric risk factor)

*“A lot of people don’t have their families in London*, *and I think that’s why you bond so quickly with your antenatal group friends*, *because you need the support*, *and you need people that are going through the same thing as you”* (IW_133, Standard, White, one obstetric risk factor)

Loneliness or relationship issues were reported similarly in both groups and were related to family. Two women specifically described lack of support with feeding or lack of family nearby:

*“I am from Australia so my family’s not here and my husband’s English but his family’s quite far away*, *so we were kind of on our own… my mum wasn’t here*, *my sister’s not here*, *like his parents*, *everyone…”* (IW_90, Standard, White, one obstetric risk factor)

*“I also think my mum was here… and she’s not an advocate for breastfeeding at all*. *Um*. *I think if she wasn’t here*, *I think I would have maybe persisted a bit more*, *and a bit longer*. *But because that my mum was here*, *I think it was one a distraction*, *and two because she didn’t advocate it*, *so she wasn’t pushing it*, *for me neither*. *And I just needed that*, *I just needed that push as well*, *but because I’d lost that confidence*.*”* (IW_194, Standard, Black, one social risk factor)

### Trust in midwives

The TNS [22] summative response scores were significantly greater in the POPPIE group compared to the standard group indicating that stronger levels of trust in midwives were reported (-4.21, -5.44 to -2.97; Mann-Whitney U, p<0.0001). When asked to write a number between 1 and 10 to rate trust on midwives, women in the POPPIE group were significantly more likely to give them a high score compared to women in the standard group (9.55 and 8.14 respectively; Mann-Whitney U, p<0.0001).

In the qualitative interviews, the majority of women in the POPPIE group reported trust in midwives with respect to acting in their best interests and being reliable and truthful, compared to fewer women in the standard group.

*“I think you can become a little bit overwhelmed*. *It was nice to have people whose opinions I trusted when we were working everything out in the early stages”*. (IW_37, POPPIE, White, one obstetric risk factor)

*“…And when they needed to act*, *they always acted very quickly*, *when it was important*. *I’m quite… quite a strong person myself*, *and to put trust in other people*, *and people you don’t know as well*, *it’s very hard for me to do…and*, *you know*, *because I trusted them*, *and I knew they knew what they were talking about…”* (IW_39, POPPIE, Black, one obstetric risk factor)

Overall, the proportion of women reporting a certain level of mistrust was higher in the standard group compared to the POPPIE group and mainly related to lack of support or midwives not available when needed for breastfeeding support:

*“When he was born*, *I have lots of pain and I can’t do nothing hardly*, *I can’t move my hands*. *I can’t do nothing*. *After midwife come*, *and said would you like try breastfeeding*? *I said yes*. *Yes*, *of course I would*! *She said*, *OK*, *I will come in a few minutes*. *Now it’s 4 years*, *she still not coming*!*”* (IW_237, Standard, White, one obstetric risk factor)

*“And then I went up to the postnatal… I was a bit upset because I still did not manage to feed him because they still did not give me to feed him and the lady said… ‘Promise me that*.*’ ‘Yeah*, *yeah*, *in the evening*, *we are going to get the baby up for you*, *so do not worry*. *Do not worry*.*’ And I was waiting for that evening and nothing*. *Nothing again…”* (IW_30, Standard, one obstetric risk factor).

### Perceptions of safety and quality of care

The perceptions of safety mean scores during the antenatal care were significantly higher in the POPPIE group compared to the standard care group indicating higher levels of perceived safety in terms of communication and consent, sufficient and trained staff, support and timely interventions (-2.01, -3.01 to 0.47; t-test; p = 0.0138). The perceptions of safety scores during the intrapartum and postnatal care were similar in both groups and ranged from 24.46 to 28.28 (indicating average-high levels of perceived safety at birth and postnatally).

Findings from the additional safety and quality related questions in the survey showed that, compared to women in the standard group, women in the POPPIE group were significantly more likely to have a particular midwife to contact when they needed during the pregnancy (98.9% vs 67.11%, chi-squared, p<0.0001) and during the postnatal period (100% vs 61.8%, chi-squared, p<0.0001). Less than 1% and nearly 36% of women in the POPPIE and standard group respectively reported they had no one to contact antenatally and postnatally. Almost 90% women in the POPPIE group were able to access their midwife via mobile phone (i.e. calls, texts) compared to 71% in the standard group. Women in the POPPIE group were also more likely to feel they were spoken in a way they could understand (100% vs 97.4%, p<0.0001) and being involved in decisions regarding the antenatal care (94.4% vs 75.0%, chi-squared, p = 0.002), intrapartum care (86.7% vs 67.1%, chi-squared, p = 0.008) and postnatal care (91.9% vs 73.6%, chi-squared, p = 0.006); whereas a third of women in the standard group would have prefer to have been more involved in decision making along the continuum care pathway. There were no significant differences between groups in the reporting of feelings that concerns raised during labour and birth were taken seriously (74.4% vs 59.2%, chi-squared, p = 0.063), or not being left alone during childbirth at a time of worries (76.7% vs 69.7%, chi-squared, p = 0.302), however clinically this is important to note.

Overall, women receiving the POPPIE model of care were more likely to receive postnatal support and advice about: feeding the baby (80.7% vs 48.7%, chi-squared, p<0.0001); handling, settling and looking after the baby (64.7% vs 35.5%, chi-squared, p<0.0001), baby’s health and progress (78.4% vs 60.5%, chi-squared, p = 0.039), their own health and recovery after the birth (78.4% vs 55.3%, chi-squared, p = 0.006) and who to contact about any emotional changes (72.7% vs 47.3%, chi-squared, p = 0.005).

During the interviews, all women in both groups described perceptions of safety (or lack of safety) and quality of care, particularly in relation to information communication and overall support. Most women in the POPPIE group reported good access to midwives for advice; information, choice and advocacy, discussion and decision making, and reported good communication with and between team members.

“You feel less scared because you’ve got that constant reassurance, and information, like they’re constantly, I could text [name of midwife] and say, you know, ‘Are my bloods back?’ and she’d text back and say, ‘Yeah all clear.’ And it’s like, great, I don’t have to wait for a doctor’s letter, it’s that kind of constant information”. (IW_175, POPPIE, White, one obstetric risk factor)

“They were very confident in the information and knowledge that they had. … and believable, and that’s what helped me… and they [midwives] definitely talk to each other about the women they have. They’re fully aware, I didn’t have to go in and, and re-talk about my situation, because they were already aware, they knew, because they spoke” (IW_39, POPPIE, Black, one obstetric risk factor)

However, more than half of women in the standard group commonly reported issues with difficult access to midwives and information, missing referrals or conflicting information by health providers:

“We had no appointment with the midwife. Nobody had seen me in the first 3 months of my pregnancy, bearing in mind that I’d had a miscarriage the previous year. Then, I was chasing them, nobody was contacting me. I was getting brushed off on the phone…” (IW_193, Standard, White, three or more obstetric/social risk factors)

“They (hospital midwives) sent an email to (community midwives) say that I’m walking around not having a midwife, and I’m high-risk pregnancy. So, a couple of days later I got a phone-call and then I saw the midwife” (IW_254_Standard, Black, two obstetric risk factors)

Most women in the POPPIE group and half of those in standard group felt being supported with various aspects of their care e.g. convenience in timing and location of appointments, practical support, reassurance, chasing up results, involvement of partners and family, personal conversations and emotional help, postnatal support and breastfeeding.

“They come here, they always come here. Which is so nice, knowing like you’ve got other kids, like it made life easier for them coming to me” (IW_123, POPPIE, White, one obstetric risk factor)

“… yeah there was a very big difference in, in the level of care, a huge difference. So, but yeah again it was just like the big things were the chasing of appointments, the reassurance, the chasing of results, like, they’d give you the results and text you the next day rather than having to wait and worrying…” (IW_190, POPPIE, White, one obstetric risk factor)

“And it’s also made me feel, when they said to me, ‘You know, you’re doing great and we’re going to discharge you, we have no concerns,’ it made me feel good as well, because, oh I’m doing a good job then, because you, no one, as a mum no one gets to tell you that really” (IW_39, POPPIE, Black, one obstetric risk factor)

“…I mean they (midwives) were very good at all, their job, at what they did… She had done her research, she had read through everything, so she did know what happened, I didn’t have to like go over it, you know, again… Um.. yeah, she put our minds at rests so that, as much as she could” (IW_109, Standard, White, one obstetric risk factor).

A few women in both groups described mixed experiences of the neonatal unit from very caring and supportive with parents to very inconsistent advice provided by different staff. Women in POPPIE and standard groups also respectively described the lack of sufficient and/or trained staff but most of them acknowledged a stretched, overworked and underfunded NHS:

“The resources are stretched so thin, and you know, it’s a real struggle I think for, for people to give you anything. I think, I think all the midwives you see in your pregnancy really want to give you the best care possible, but they’re just so over-worked.” (IW_171, POPPIE, White, one obstetric risk factor)

“…I know this one thing as well, they’re understaffed a lot, so I can understand when they’re busy and stuff like that they don’t have time to read the book…” (IW_254, Standard, Black, two obstetric risk factors)

Half of women in the POPPIE group and a few women in the standard groups perceived interventions were timely given or followed up (e.g. test results and treatment if needed, referrals, epidural for pain relief, emergency caesarean sections) compared to less than a quarter of women in respective groups who perceived no early midwifery support or experienced delays in inductions, lack of referrals, resources or scan appointments.

“I would contact [name of midwife] occasionally by text if say she’d done a urine test and sent it off and would text me saying it was fine. Or just to check if, you know, I’d had a scan just to see how that was, I’d text and say they were all fine.” (IW_197, POPPIE, Black, one obstetric risk factor)

“She went, and what’s the physio, is the physio helping? It was like, I haven’t seen any physio! I was told, she told me there was at 6 week or 8 weeks wait for physio. And she didn’t refer me…” (IW_193_Stan, White, three or more obstetric/social risk factors)

### Control during childbirth

The LAS [25] scores reported in the survey by women in both groups ranged from 49 to 53, with a mean score of 51.46 reflecting moderate control during childbirth. There were no significant differences in mean LAS scores between women allocated to the POPPIE group and women allocated to standard maternity care (-1.42, -5.37 to 2.52; t = -0.7120, p = 0.4775).

About two thirds of women interviewed in both groups described experiences of control (or lack of) during labour. Overall, women in the POPPIE group tended to report more positive emotions (e.g. confidence in oneself and others, empowerment, calm, being with people they cared about such as partner, family and midwives):

“I was calm the whole way through. I just, it, I was just like … right, that’s another contraction gone, cool, one more. Another one gone. That’s one less, right, one less. Cool. Breathe. Yeah, next, gone. Because it’s just like if you count them down like, you know, right, that’s one less that you’re going to have.” (IW_118, POPPIE, White, one obstetric risk factor)

“I don’t remember being told to push or anything like that, it all sort of just happened… But I think … I think it’s like the biggest thing for me with the POPPIE experience was that I didn’t feel any fear and I think that without the fear there it would just, you just feel a lot safer anyway… (IW_171, POPPIE, White, one obstetric risk factor)

and negative emotions (e.g. things not happened as planned, anxious about concerns, powerless for failed progress):

“I think I was a little bit demoralised and I was a bit like, you know, bloody hypnobirthing, it’s, you know, they said it wouldn’t hurt!” (IW_23, POPPIE, White, one obstetric risk factor)

In comparison, women in the standard group tended to report less of both positive and negative emotions:

“I feel, I felt very, very supported and very, very cared for the whole way through, like, there wasn’t, there wasn’t a moment where I felt like, um, oh, I wasn’t worried at any point, I felt so confident in the care that I was having. I, I felt very, very valued and very, very cared for the whole way through.” (IW_194, Standard, Black, one social risk factor).

“So I was really anxious that something could still happen to me or her, that hadn’t been picked up, because they weren’t in control of the situation. Um, so yeah, if I was listen-, if there was more control, she was born in a more controlled way, I might not have felt (that way)…” (IW_138, Standard, White, one obstetric risk factor).

### Bonding

The MBIS [26] scores reported by women in the survey did not differ between groups and ranged from 1.37 to 2.11 with a mean difference score of 0.45 (-0.28 to 1.20), p = 0.2085 indicating overall very good bonding and positive feelings for their child in the first few weeks.

Nearly a third of women in both groups specifically described experiences of bonding during interviews. Loving, joyful and protective feelings towards their babies were reported by most women in both groups:

“He was born, he looked straight at me and … we were kind of, ‘Oh he’s lovely,’… I feel like the motherly instinct really kicked in the minute he was born…” (IW_39, POPPIE, Black, one obstetric risk factor)

Whereas disappointment, resentfulness and neutrality or feeling nothing were described by only one woman who was in the POPPIE group and three women in the standard group:

“I couldn’t hold him, I was throwing up, I was so out of it, I was, like speaking I didn’t know what I was saying, like for hours, I mean hours I couldn’t hold him. It was horrible. It was awful… And, that’s just really, it’s just a bit upsetting”. (IW_90, POPPIE, White, one obstetric risk factor).

“Because I, because of feeding, because I couldn’t bond with him. Because I’m like, oh I’ve brought you into the world, and I’m not sure I want you. And, how can I feel that way about you when it’s not your fault? It, and then, and people like are saying, oh yeah, get out there, go and meet other mums, and I’m like the last thing I want to do is see another mum, who’s there happy there with their kid, and I c-, can’t, you know, do, go to the baby without crying…” (IW_109, Standard, White, one obstetric risk factor)

### Quality of life

There were no differences in the self-report measurement of physical and mental health domains between both groups (Mann-Whitney U; p = 0.3610 and p = 0.8461 respectively). Means for the GPH ranged from 15.43 in the POPPIE group to 15.80 in the standard group, while means for the GMH ranged from 14.65 in the POPPIE group to 14.76 in the standard group. Overall women’s GPH and GMH T-scores indicated good physical and fair mental symptoms and function, less than half a SD worse than the (US) general population average.

Health and quality of life were reported in interviews by half of women in both groups. Positive and negative mental health was similarly reported by women in both groups (e.g. relaxation and stress, feelings of anxiety or depression):

“Having the POPPIE team helped me with my anxiety. Hugely. I can’t tell you how much. Absolutely, because, you know, I wasn’t, if you’re familiar with somebody and you sort of know where their, what their ethos is, you know where their advice is coming from, so it felt a lot more, I don’t know, I just felt like I could trust the advice a lot more…” (IW_37_POP, White, one obstetric risk factor).

“Well I suffered with anxiety quite a few times and every time I spoke to [name of midwife M] and she sort of said, ‘Right, OK, well let’s … you know, what’s your concerns?’ and we talked through them, and it … and I felt so much better.” (IW_26, POPPIE, White, three or more obstetric/social risk factors).

Negative physical health such as fatigue and pain were mainly reported by women in the standard group (e.g. infections, urine incontinence, symphysis pubis dysfunction, coccyx pain):

“By the time I broke down in the hospital, I couldn’t, couldn’t walk properly… I was in so much pain, and the midwife from the community, when I said to her my SPD is coming, then said to me, just come here for your appointments…” (IW_138_Standard, White, three or more obstetric/social risk factors)

“… I was feeling really rough. I just thought right okay, I’m run down, tired, sweating, just not feeling well. But I had a chest infection and didn’t realise” (IW_281_Standard, Black, two obstetric risk factors)

whereas women in the POPPIE group tended to report more positive physical health (e.g. healthy pregnancy, regular walking).

“And there were no complications or stress with him beforehand, or no blood pressure, no nothing like that, everything was fine” (IW_118, POPPIE, White, one obstetric risk factor)

In summary, experiences and perceptions of trust, quality and safety were improved among women in the POPPIE group participating in the postnatal survey compared to those in the standard group.

## Discussion

This study reports results of a postnatal survey and qualitative interviews exploring the experiences of care among women at increased risk of PTB participating in the POPPIE pilot trial and receiving either POPPIE continuity of care or standard maternity care. Overall, survey respondents in the POPPIE group were more likely to report greater trust in midwives and perceptions of safety and quality care than survey respondents in the standard group; however, there were no differences between groups in reported social support, control during labour, bonding and quality of life after birth. Results from qualitative interviews provided insight and depth into many of these findings, with women in the POPPIE group reporting more positive experiences of bonding towards their babies and more positive physical health postnatally.

The significant positive impact of midwifery continuity of care on perceptions of trust and safety in relation to many aspects of maternity care (e.g. access, communication, choice, decision making, advice and support) is not surprising. Both are hypothesised mechanisms linking continuity models with improved experiences and outcomes; they ensure a safe care model based on a relationship of mutual trust and respect in line with the woman’s decision in which women are more likely to disclose risks or potentially harmful behaviours (e.g smoking, drinking), be prepared to trust advice and engage in self-care activities and accept referrals for support [36, 37] Although there were no significant differences between groups in the reporting of concerns raised during labour and birth were taken seriously, or being left alone during childbirth at a time of worries, clinically the size of the difference warrants further exploration in future research. The significant differences in advice and support women in the POPPIE group received in the immediate and late postnatal periods are important and aligned with findings from a previous study [38]. Future research should also explore if this may partially explain why some women interviewed in the continuity group reported more bonding and positive physical health following birth.

There is mixed evidence from previous literature in relation to women’s experiences of control during childbirth. One study used a three‐point scale to measure perceptions of control and found that continuity was associated with women feeling more prepared for labour [39]. When using adapted questionnaires to assess experience of childbirth in two studies, women receiving continuity were more positive about their overall experiences than women receiving standard care and felt more in control during labour, were prouder of themselves, less anxious, and more likely to have a positive experience of pain [40, 41]. Our findings, however, are similar to others in that no differences in control of labour and birth experiences between women in the continuity and standard groups [42, 43]. Although women in the POPPIE group tended to report both more positive and negative emotions than women in standard group in relation to control during childbirth, which are potentially associated with fulfilment or lack of fulfilment of their expectations [44].

Maternity services should be able to provide women and their partners a safe transition to parenthood to have a positive and life enhancing experience that sets down important foundations for healthy living [45]. It is important to note that extra support, advice and parenting education during the postnatal period were emphasised by many interviewed women in the POPPIE group. They felt their partners were also involved and supported throughout the childbearing journey and described the midwife as someone being “part of the family”; and this involvement and support to women and their partners and families can have a significant public health impact on their future social, emotional and intellectual development [46]. Although extra support in the postnatal period was highly valued, much research is needed to understand why continuity of care improves some aspects of experiences of maternity care and not others and which aspects are important to whom.

In conclusion, compared with standard maternity care, women receiving continuity of midwifery care reported significantly improved experiences of trust, safety and quality of care. Although there was a differential survey response rate and interview acceptance rate between both groups, data was stratified to explore any differences arising from this in relation to exposure variables that might affect women’s experiences. Interview data came from a less diverse demographic sample compared to the survey data and this possibly limits richness and breath in the data, particularly for some sub-groups of women. The main pilot trial included a high-risk population with more than one quarter of women in both groups having one or more pre-existing medical conditions and multiple obstetric and social risk factors for PTB [14]. Since people who are socially disadvantaged and have complex needs, and those from ethnic minority groups are less likely to engage in research follow up [47], further investigations should focus on community participatory research working in partnership with women with more complex needs who have multiple clinical and social risk factors. Our findings inform current NHS maternal policy on models of midwifery continuity of care which aims to improve safety and quality of care for women who are socially disadvantaged and from ethnic minorities communities [9]. Still much research is needed to understand experiences of care and potential mechanisms of continuity models (e.g. safety, quality, engagement) among low and mixed risk women, particularly those with complex social factors and vulnerability such as women who find services hard to access.

## Supporting information

S1 File Internal consistency of the Social Support Scale (SSS) [19] in women at risk of preterm birth responding to the postnatal survey. (DOCX)

S2 File Structural validity and internal consistency reliability of 5 item Trust in Midwives scale in women at risk of preterm birth responding to the postnatal survey (adapted version of the 5-item Trust in Nurses Scale TNS) [20]. (DOCX)

S3 File Internal consistency and factor structure of 7 item perceptions of safety scale in women at risk of preterm birth responding to the postnatal survey (shortened version of the 13-item scale in the Perceptions of Safety Measurement Questionnaire [21]). (DOCX)

S4 File Details of scales: numbers, proportions and statistical tests. (DOCX)

S5 File Interview topic guide for the POPPIE pilot. (DOCX)
