# Supplementary material for: Experiences of maternity care among women at increased risk of preterm birth receiving midwifery continuity of care compared to women receiving standard care: Results from the POPPIE pilot trial
Source: PLoS One. 2021 Apr 21;16(4):e0248588. doi: 10.1371/journal.pone.0248588 (PMC8059847; doi:10.1371/journal.pone.0248588)
Supplement: S1 File — (DOCX) [file pone.0248588.s001.docx]

**S1 File: Internal consistency of the Social Support Scale (SSS) [20] in women at risk of preterm birth responding to the postnatal survey**

**Cronbach’s alpha test**

Cronbach’s alpha test scale and mean inter-item correlations were used to examine internal consistency reliability of each item and overall scale (0.77):

| Question 1 | I have no one to share my feelings with |
| --- | --- |
| Question 2 | My partner provides the emotional support I need |
| Question 3 | There are other mothers with whom I can share my experiences |
| Question 4 | I believe in moments of difficulty my neighbors would help |
| Question 5 | I'm worried that my partner might leave |
| Question 6 | There is always someone with whom I can share my happiness and excitement about my baby |
| Question 7 | If I feel tired, I can rely on my partner to take over* |
| Question 8 | If I was in financial difficulty, I know my family would help if they could |
| Question 9 | If I was in financial difficulty, I know my friends would help if they could |
| Question 10 | If all else fails I know the state will support and assist me |

**
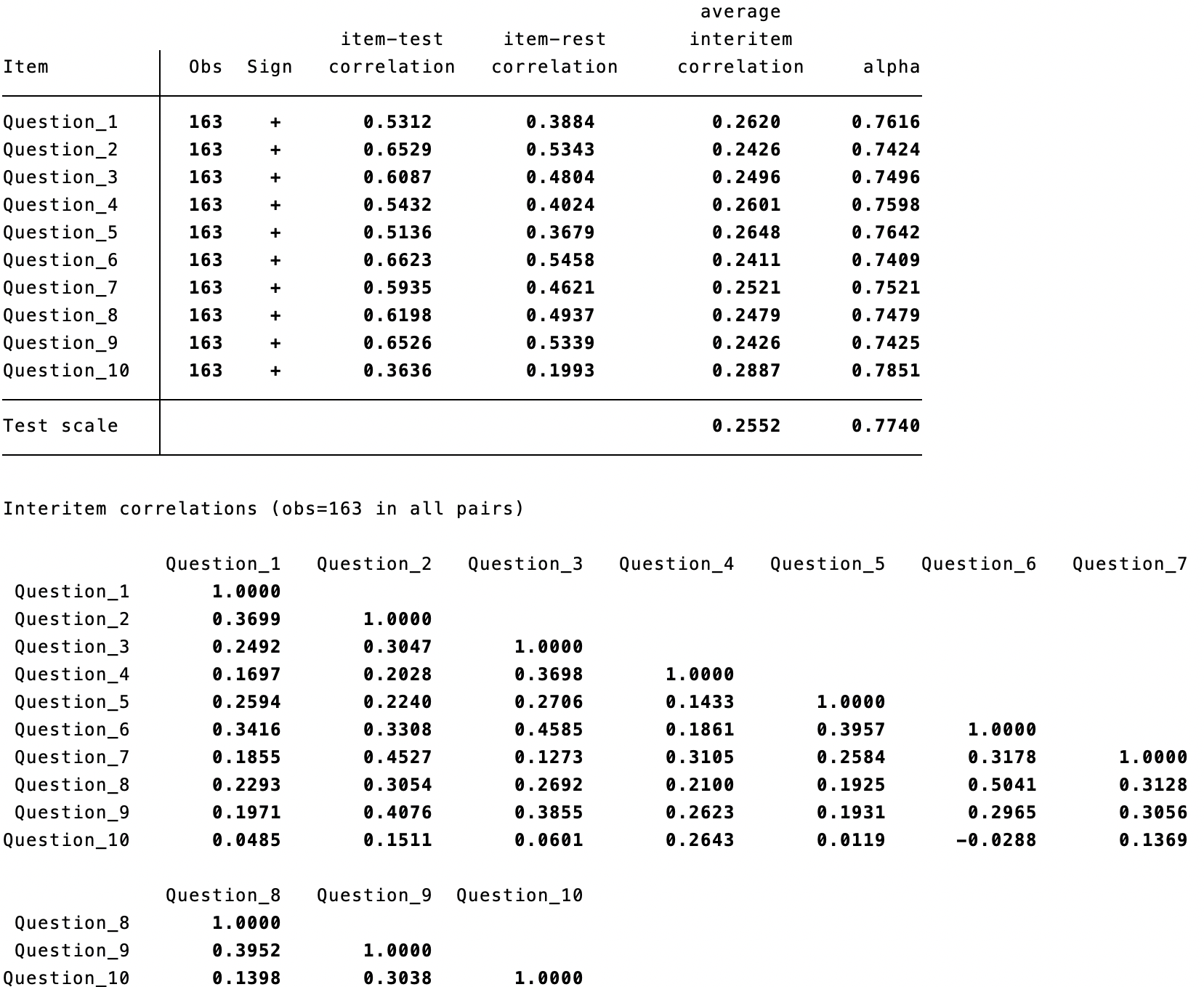
**
