# Supplementary material for: Experiences of maternity care among women at increased risk of preterm birth receiving midwifery continuity of care compared to women receiving standard care: Results from the POPPIE pilot trial
Source: PLoS One. 2021 Apr 21;16(4):e0248588. doi: 10.1371/journal.pone.0248588 (PMC8059847; doi:10.1371/journal.pone.0248588)

**S2 File: Structural validity and internal consistency reliability of 5 item Trust in Midwives scale in women at risk of preterm birth responding to the postnatal survey (adapted version of the 5-item Trust in Nurses Scale TNS) [21]**

**Data preparation**

|  | Question 1 | Question 2 | Question 3 | Question 4 | Question 5 | Global item |
| --- | --- | --- | --- | --- | --- | --- |
| Trust in Nurses Scale (TNS) | How often were your nurses there when you needed them? | How often did you believe that your nurses were acting in your best interest? | How often did you trust what your nurses told you? | How often did your nurses do what they said they would do? | How often did your nurses provide accurate information about the cancer? | Please rate how much you trusted your nurses on a scale from 1 (no trust at all) to 10 (trust as much as possible) |
| Adapted TNS for Midwives | How often were your midwives there when you needed them? | How often did you believe that your midwives were acting in your best interest? | How often did you trust what your midwives told you? | How often did your midwives do what they said they would do? | How often did your midwives provide accurate information about the pregnancy? | Please rate how much you trusted your midwives on a scale from 1 (no trust at all) to 10 (trust as much as possible) |

**Principal components analysis (PCA)**


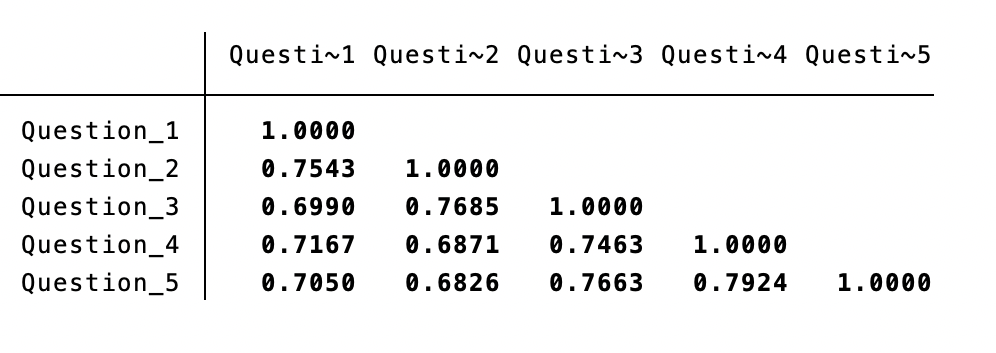


**Correlation matrix (page 2)**


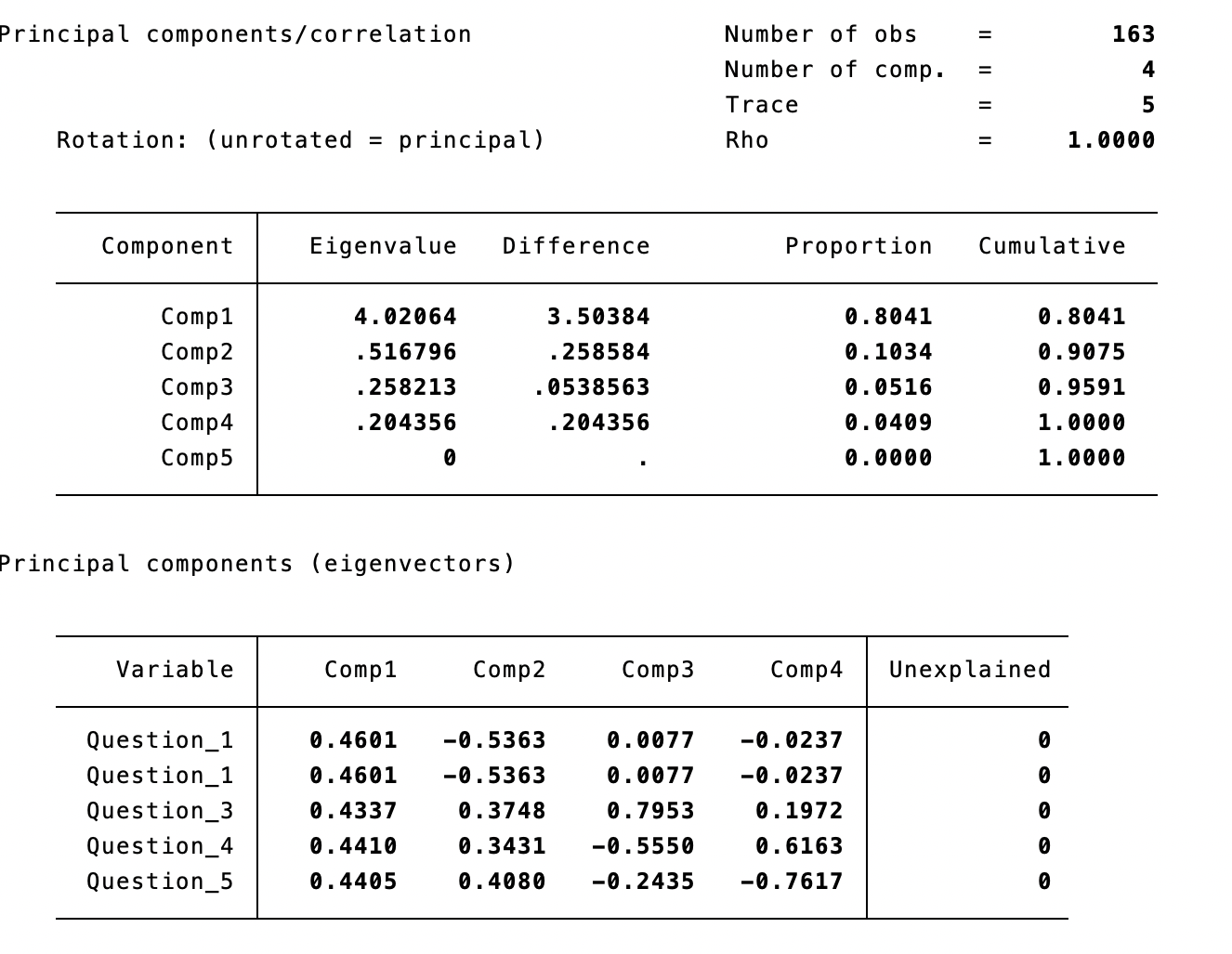


**Scree plot for eigenvalues**


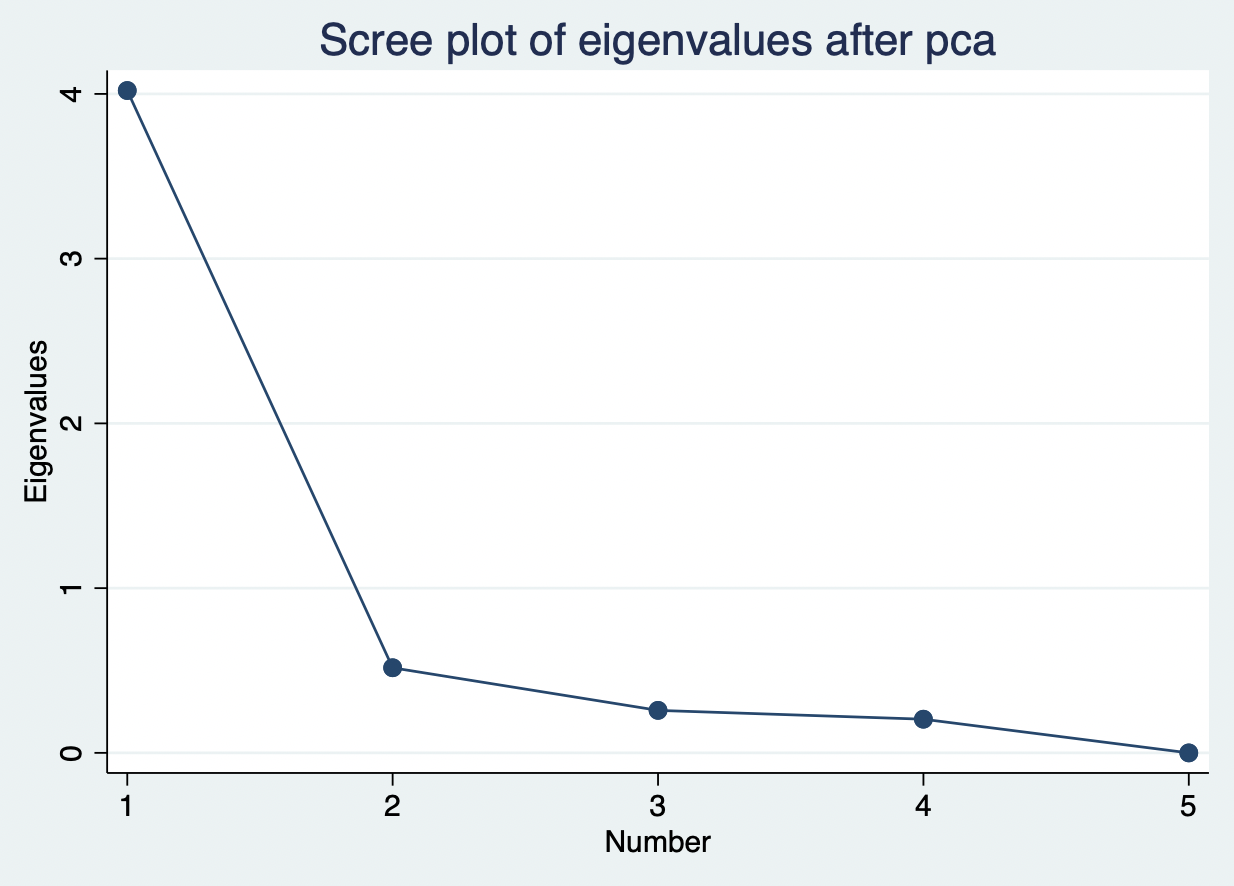


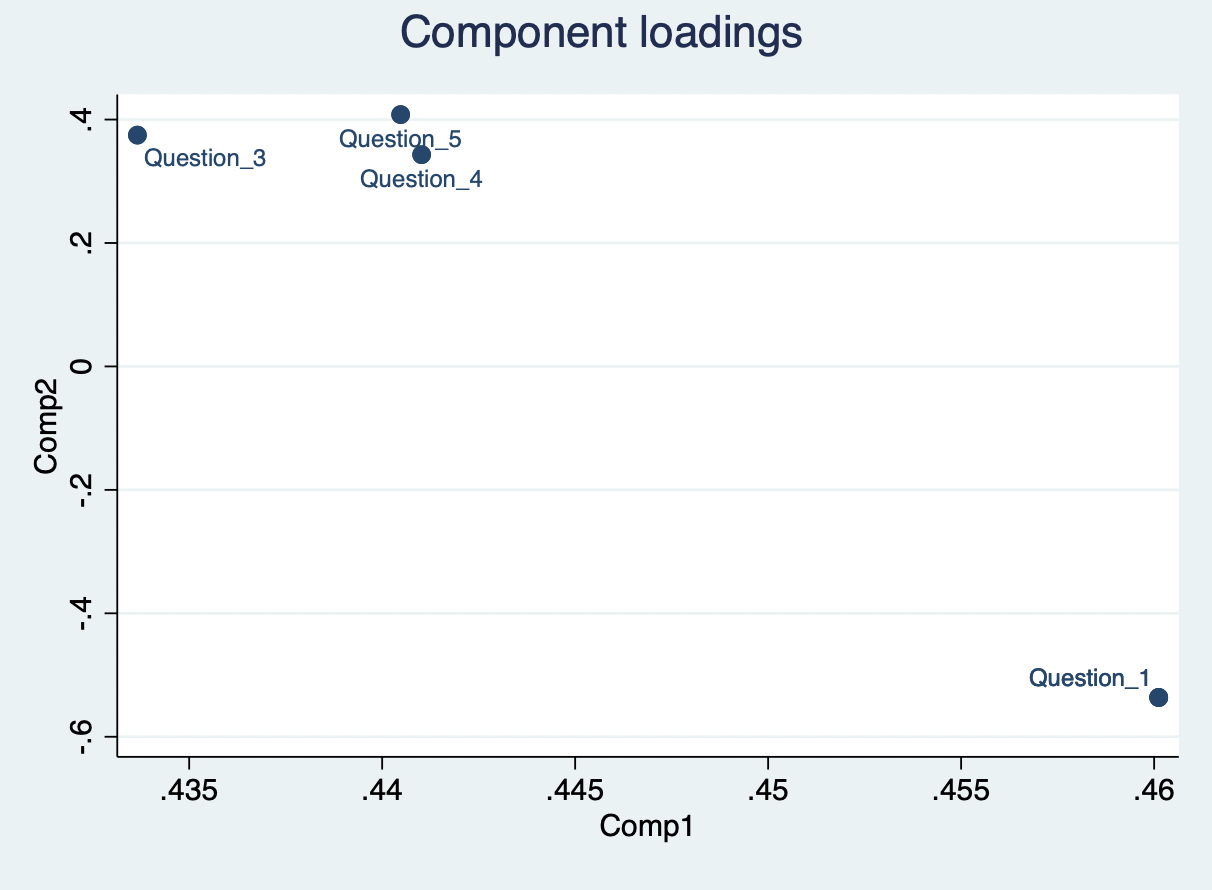
**Factor loading plot**

**Prediction of numbers for this model**

**
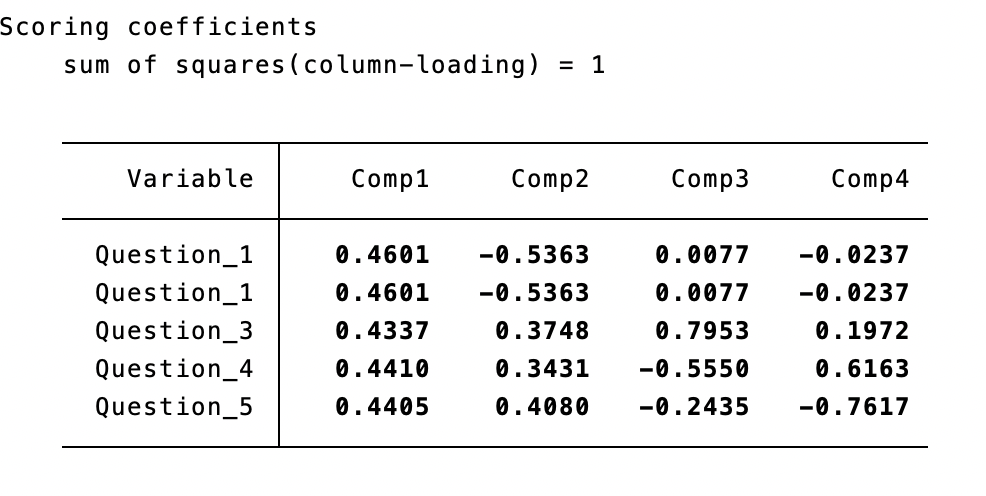
**

**Cronbach’s alpha test**


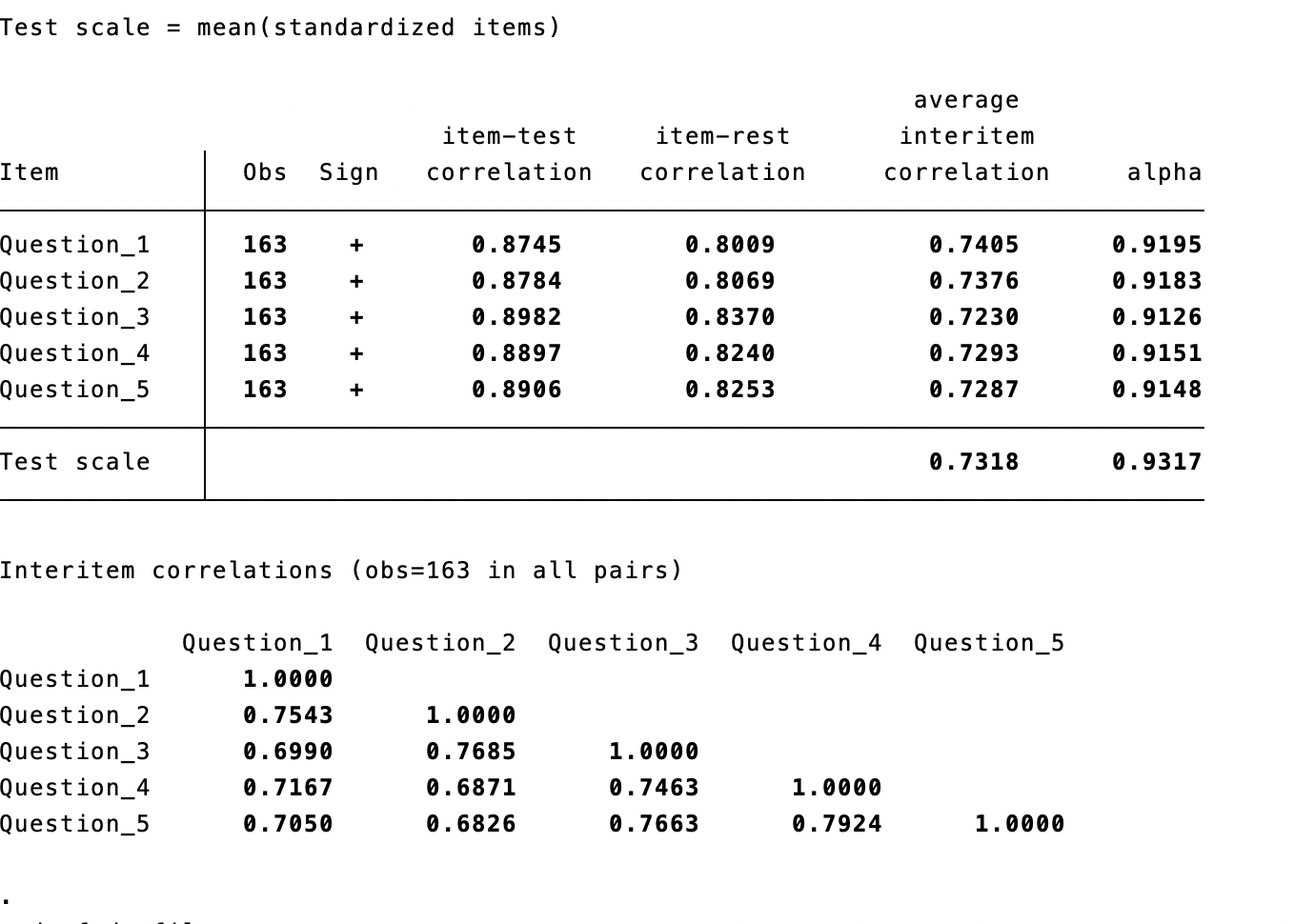

Supplement: S2 File — (DOCX) [file pone.0248588.s002.docx]
