# Supplementary material for: Experiences of maternity care among women at increased risk of preterm birth receiving midwifery continuity of care compared to women receiving standard care: Results from the POPPIE pilot trial
Source: PLoS One. 2021 Apr 21;16(4):e0248588. doi: 10.1371/journal.pone.0248588 (PMC8059847; doi:10.1371/journal.pone.0248588)

**S3 File: Internal consistency and factor structure of 7 item perceptions of safety scale in women at risk of preterm birth responding to the postnatal survey (shortened version of the 13-item scale in the Perceptions of Safety Measurement Questionnaire [22])**

**Data preparation**

| Participant ID | Question 1  I could have a member of my family or close friend for support when I wanted | Question 2  My consent was obtained before a test, an investigation or an emergency procedure | Question 3  Test and procedures were carried out when staff said they would be | Question 4  There were always enough staff to care for me | Question 5  Staff were familiar with the equipment and procedures | Question 6  If applicable, I was given information about my medication in a way I could understand | Question 7  If applicable, My discharge and my babies were well planned |
| --- | --- | --- | --- | --- | --- | --- | --- |
|  |  |  |  |  |  |  |  |
|  |  |  |  |  |  |  |  |


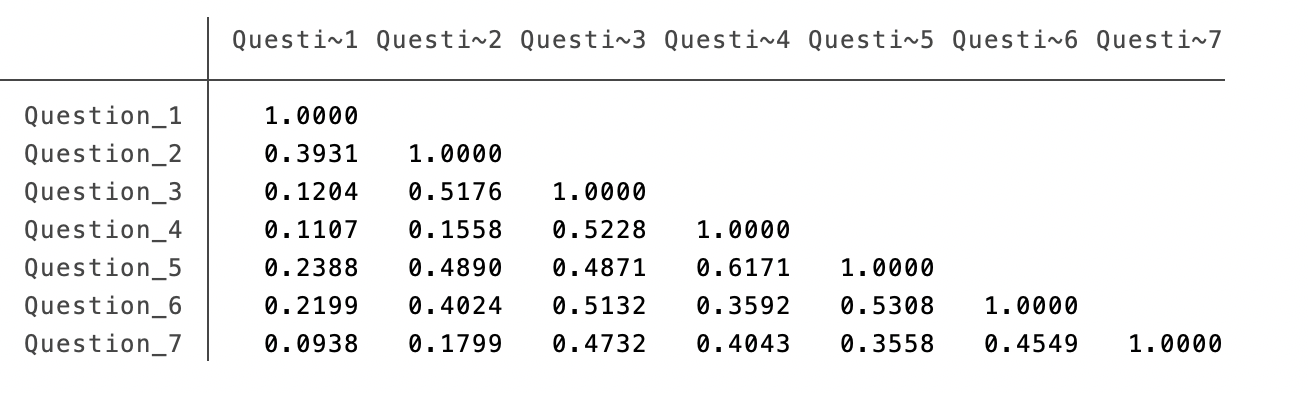
**Principal components analysis (PCA)**

**Correlation matrix (page 2)**


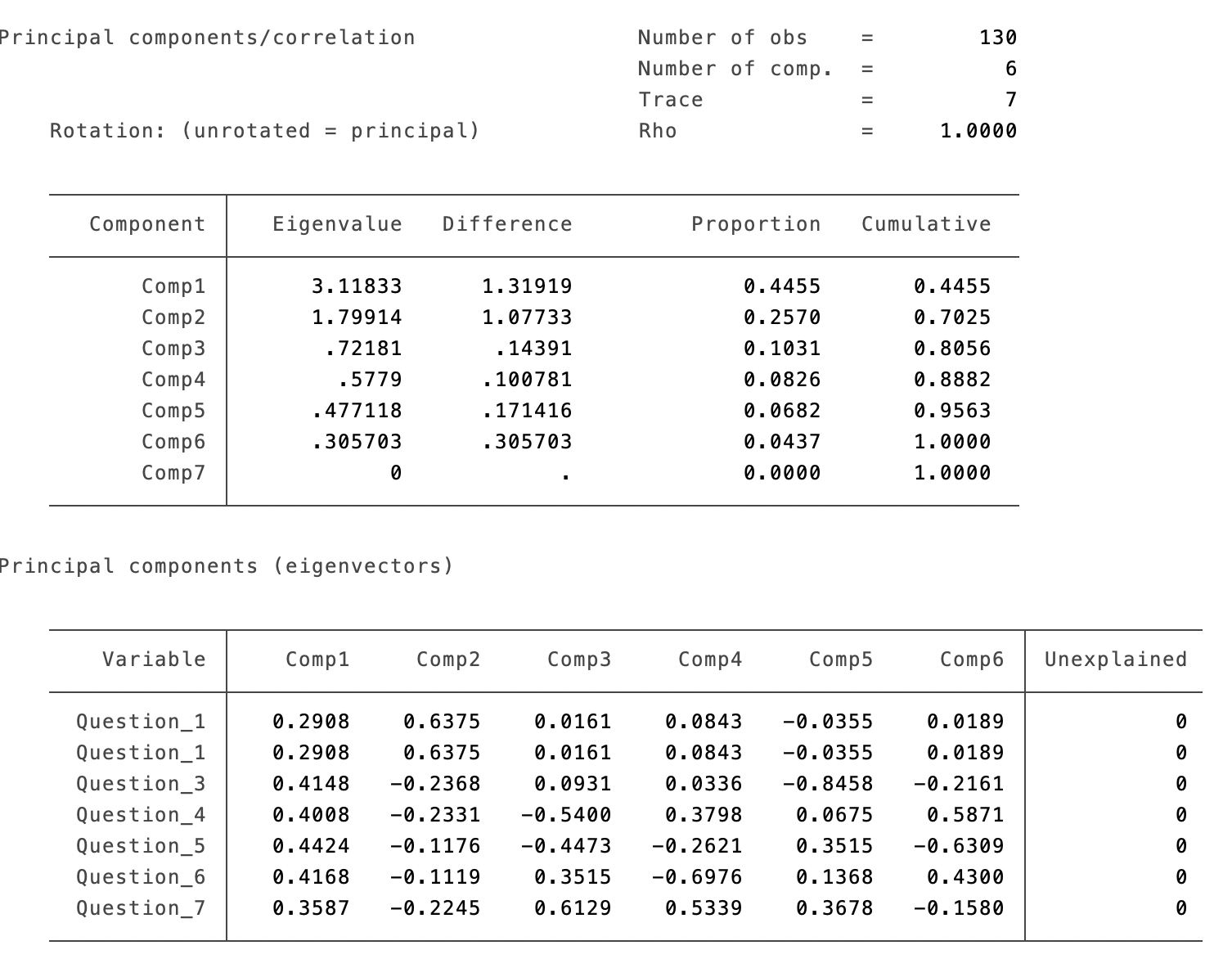


**Scree plot for eigenvalues**


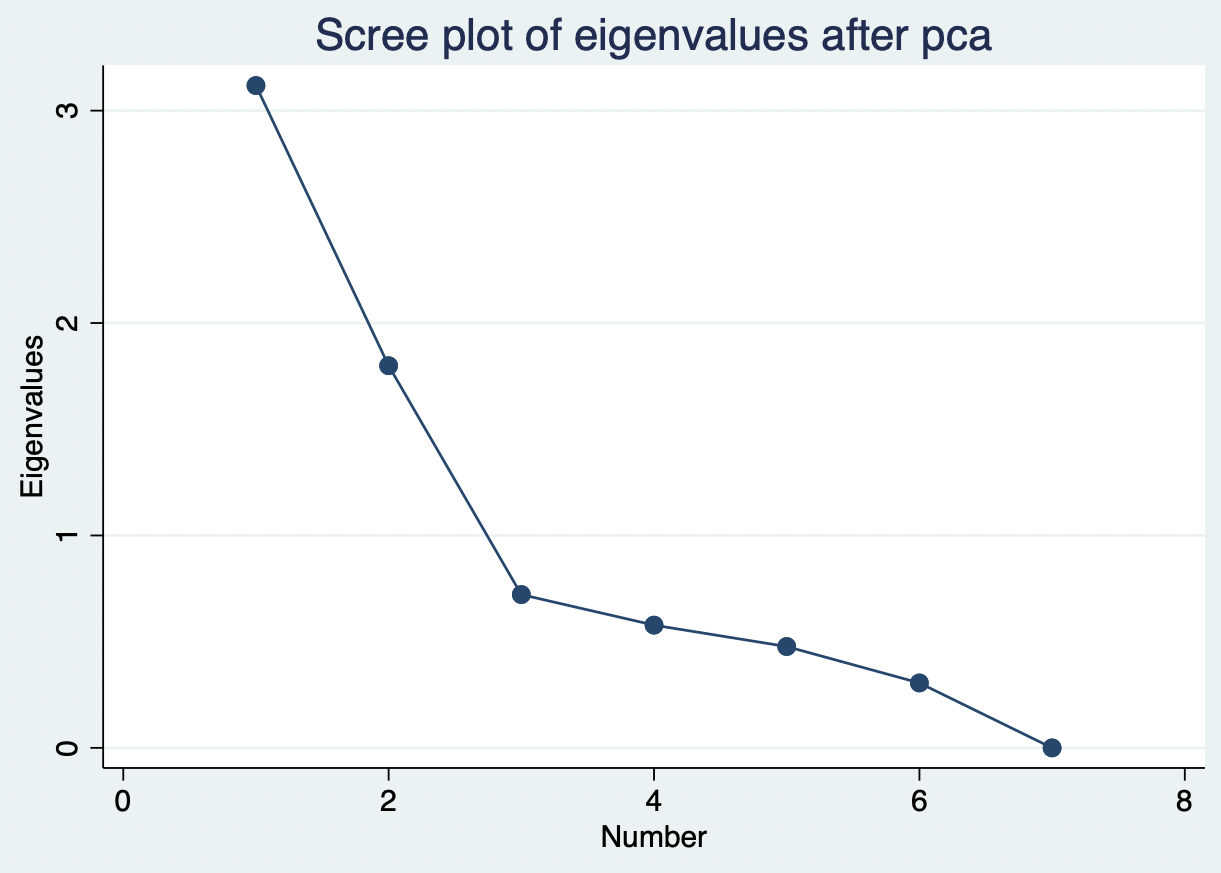


**Factor loading plot**


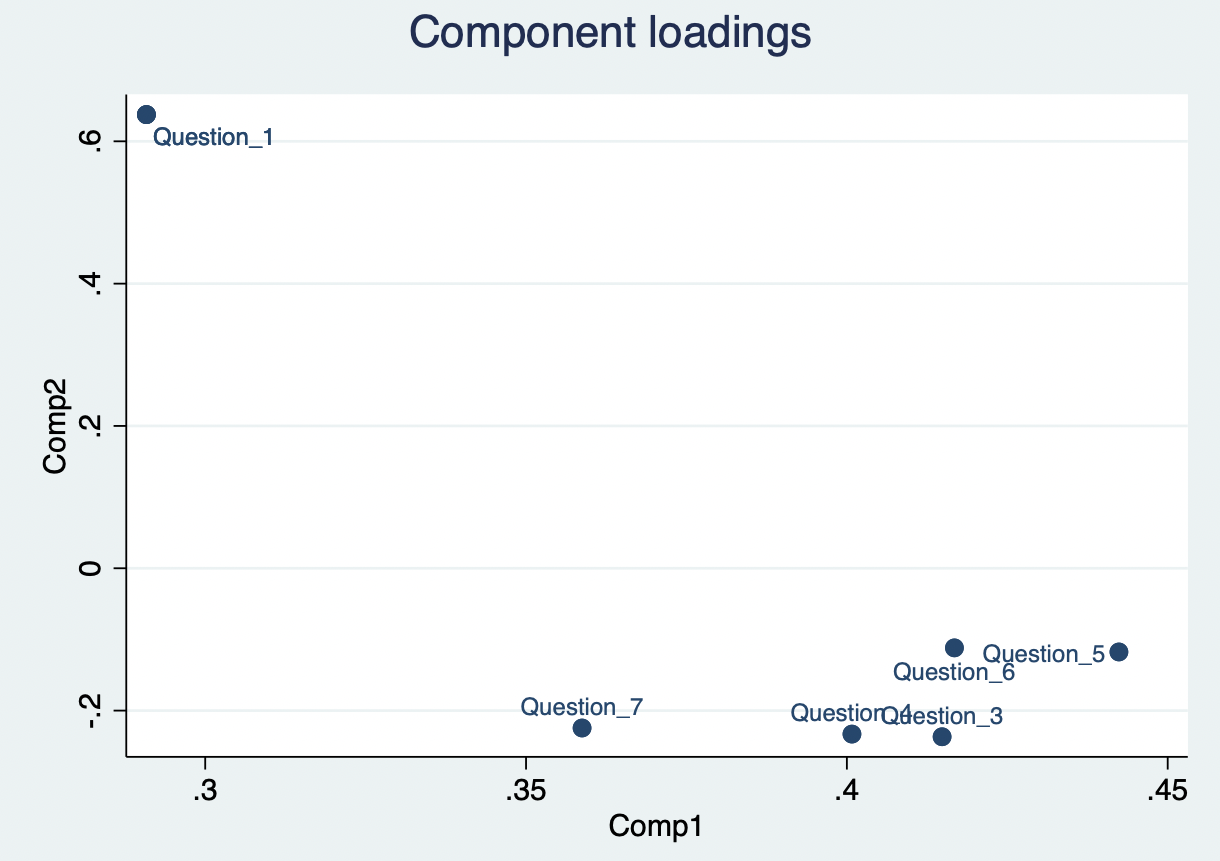


**Predicting numbers**


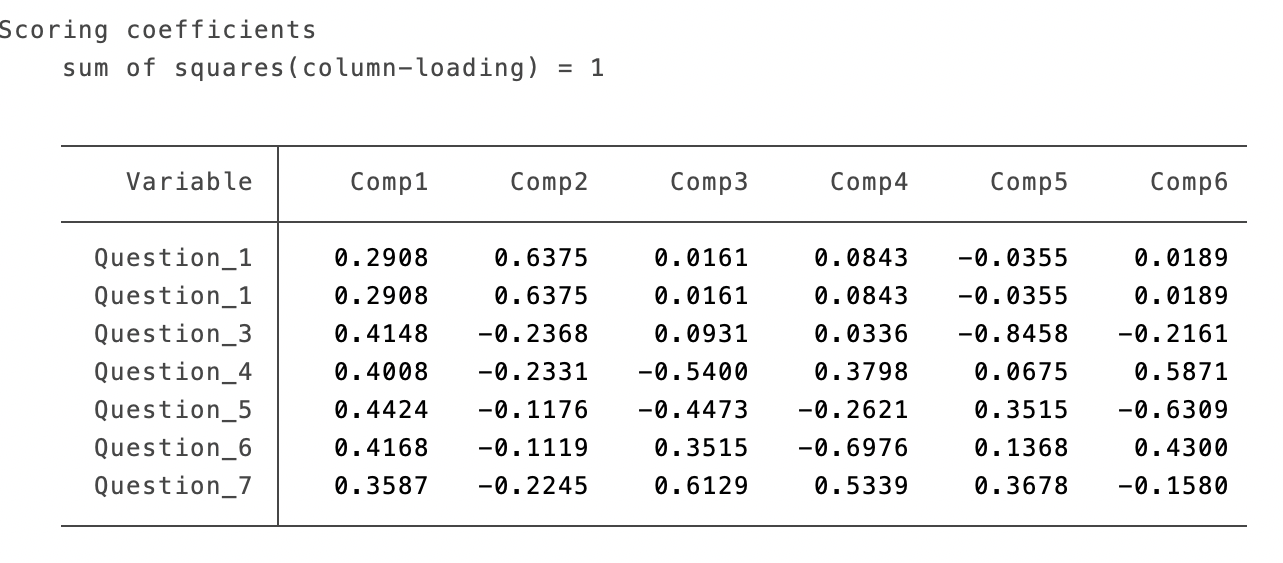


**Cronbach’s alpha test**


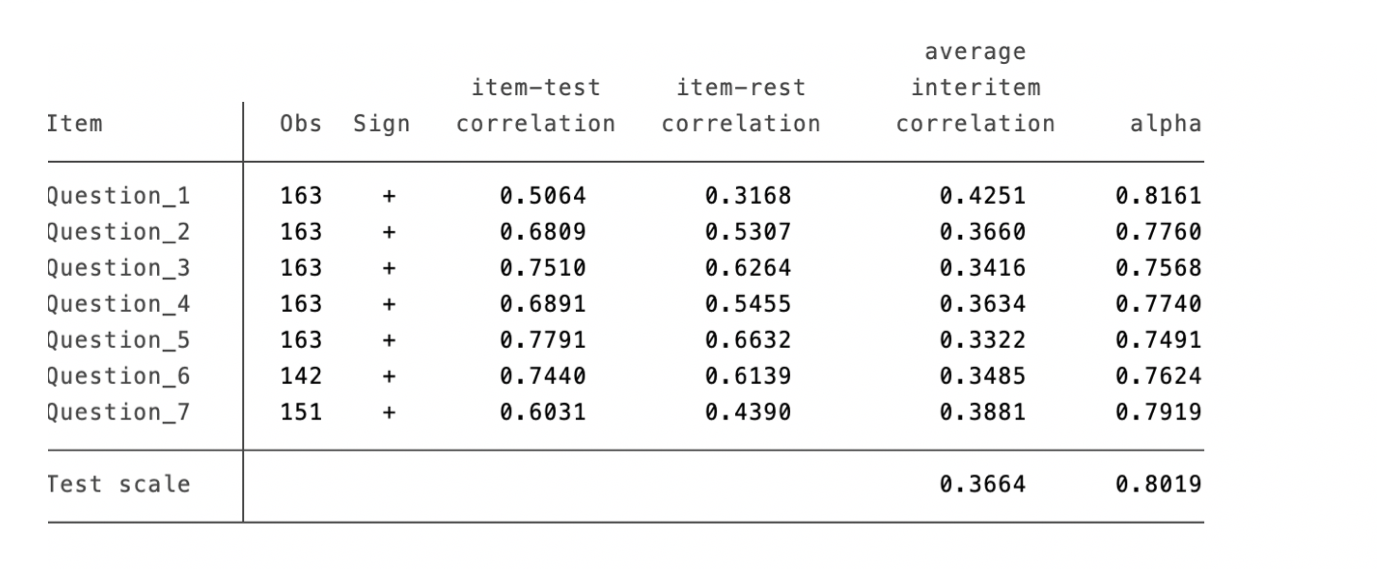


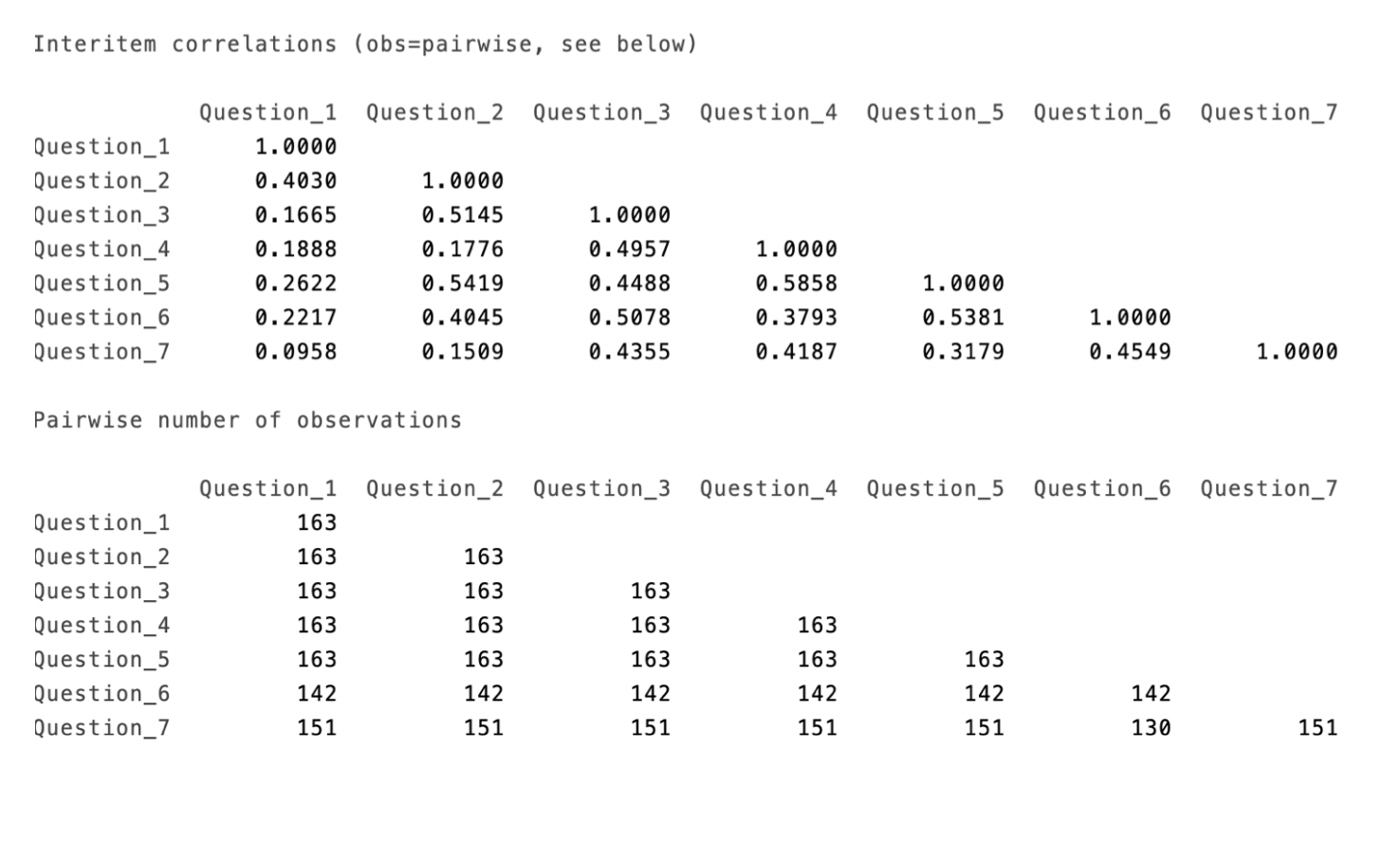

Supplement: S3 File — (DOCX) [file pone.0248588.s003.docx]
