# Supplementary material for: Experiences of maternity care among women at increased risk of preterm birth receiving midwifery continuity of care compared to women receiving standard care: Results from the POPPIE pilot trial
Source: PLoS One. 2021 Apr 21;16(4):e0248588. doi: 10.1371/journal.pone.0248588 (PMC8059847; doi:10.1371/journal.pone.0248588)
Supplement: S4 File — (DOCX) [file pone.0248588.s004.docx]

**S4 File: Details of scales: numbers, proportions and statistical tests.**

| **Social Support Scale (SSS) The following statements are about the help and support you have:** | **POPPIE**  **n (%)** | **Standard**  **n (%)** | *Total* |
| --- | --- | --- | --- |
| I have no one to share my feelings with  this is exactly how I feel (4)  this is often how I feel (3)  this is how I sometimes feel (2)  I never feel this way (1)  *Total* | 0 (0.00)  1 (1.15)  14 (16.09)  72 (82.76)  87 (100.00) | 1 (1.32)  2 (2.63)  20 (26.32)  53 (69.74)  76 (100.00) | 1 (0.61)  3 (1.84)  34 (20.86)  125 (76.69)  163 (100.00) |
| My partner provides the emotional support I need  this is exactly how I feel (1)  this is often how I feel (2)  this is how I sometimes feel (3)  I never feel this way (4)  *Total* | 50 (57.47)  19 (21.84)  13 (14.94)  5 (5.75)  87 (100.00) | 44 (57.89)  16 (21.05)  11 (14.47)  5 (6.58)  76 (100.00) | 94 (57.67)  35 (21.47)  24 (14.72)  10 (6.13)  163 (100.00) |
| There are other mothers with whom I can share my experiences  this is exactly how I feel (1)  this is often how I feel (2)  this is how I sometimes feel (3)  I never feel this way (4)  *Total* | 43 (49.43)  22 (25.29)  15 (17.34)  7 (8.05)  87 (100.00) | 30 (39.47)  20 (26.32)  17 (22.37)  9 (11.84)  76 (100.00) | 73 (44.79)  42 (25.77)  32 (19.63)  16 (9.82)  163 (100.00) |
| I believe in moments of difficulty my neighbors would help  this is exactly how I feel (1)  this is often how I feel (2)  this is how I sometimes feel (3)  I never feel this way (4)  *Total* | 22 (25.29)  11 (12.64)  29 (33.33)  25 (28.74)  87 (100.00) | 16 (21.05)  20 (26.32)  16 (21.05)  24 (31.58)  76 (100.00) | 38 (23.31)  31 (19.02)  45 (27.61)  49 (30.06)  163 (100.00) |
| I'm worried that my partner might leave  this is exactly how I feel (4)  this is often how I feel (3)  this is how I sometimes feel (2)  I never feel this way (1)  *Total* | 3 (3.45)  1 (1.15)  7 (8.05)  76 (87.36)  87 (100.00) | 3 (3.95)  3 (3.95)  6 (7.89)  64 (84.21)  76 (100.00) | 6 (3.68)  4 (2.45)  13 (7.98)  140 (85.89)  163 (100.00) |
| There is always someone with whom I can share my happiness and excitement about my baby  this is exactly how I feel (1)  this is often how I feel (2)  this is how I sometimes feel (3)  I never feel this way (4)  *Total* | 66 (75.86)  14 (16.09)  4 (4.60)  3 (3.45)  87 (100.00) | 59 (77.63)  13 (17.11)  1 (1.32)  3 (3.95)  76 (100.00) | 125 (76.69)  27 (16.56)  5 (3.07)  6 (3.68)  163 (100.00) |
| If I feel tired, I can rely on my partner to take over*  this is exactly how I feel (1)  this is often how I feel (2)  this is how I sometimes feel (3)  I never feel this way (4)  *Total* | 41 (47.13)  22 (25.29)  13 (14.94)  11 (12.64)  87 (100.00) | 37 (48.68)  23 (30.26)  13 (17.11)  3 (3.95)  76 (100.00) | 78 (47.85)  45 (27.61)  26 (15.95)  14 (8.59)  163 (100.00) |
| If I was in financial difficulty, I know my family would help if they could  this is exactly how I feel (1)  this is often how I feel (2)  this is how I sometimes feel (3)  I never feel this way (4)  *Total* | 70 (80.46)  5 (5.75)  6 (6.90)  6 (6.90)  87 (100.00) | 62 (81.58)  7 (9.21)  5 (6.58)  2 (2.63)  76 (100.00) | 132 (80.98)  12 (7.36)  11 (6.75)  8 (4.91)  163 (100.00) |
| If I was in financial difficulty, I know my friends would help if they could  this is exactly how I feel (1)  this is often how I feel (2)  this is how I sometimes feel (3)  I never feel this way (4)  *Total* | 39 (44.83)  23 (26.44)  19 (21.84)  6 (6.90)  87 (100.00) | 40 (52.63)  11 (14.47)  17 (22.37)  8 (10.53)  76 (100.00) | 79 (48.47)  34 (20.86)  36 (22.09)  14 (8.59)  163 (100.00) |
| If all else fails I know the state will support and assist me  this is exactly how I feel (1)  this is often how I feel (2)  this is how I sometimes feel (3)  I never feel this way (4)  *Total* | 20 (22.99)  10 (11.49)  37 (42.53)  20 (22.99)  87 (100.00) | 20 (26.32)  7 (9.21)  20 (26.32)  29 (38.16)  76 (100.00) | 40 (24.54)  17 (10.43)  57 (34.97)  49 (30.06)  163 (100.00) |
|  | Mean [95% CI] | Mean [95% CI] | Mean Diff [95% CI] |
| **SSS score** | 17.82 (5.91)  [16.60 to 19.04] | 18.07 (4.62)  [17.05 to 19.10] | 0.15  [-1.35 to 1.85] |
| t=0.3092, df=161, p=0.7576 | | | |

| **Trust in healthcare providers has been related to better compliance with optimal care, self-care and other important outcomes. We would like to explore your feelings and your trust on the midwives involved in your maternity care.** | **POPPIE**  **n (%)** | **Standard**  **n (%)** | *Total* |
| --- | --- | --- | --- |
| How often were your midwives there when you needed them?  never (1)  rarely (2)  some of the time (3)  a good bit of the time (4)  usually (5)  always (6)  *Total* | 0 (0.00)  0 (0.00)  1 (1.15)  3 (3.45)  18 (20.69)  65 (74.71)  87 (100.00) | 2 (2.60)  6 (7.79)  6 (9.09)  9 (11.69)  22 (28.57)  31 (40.26)  76 (100.00) | 2 (1.22)  6 (3.66)  7 (4.88)  12 (7.32)  40 (24.39)  96 (58.54)  163 (100.00) |
| How often did you believe that your midwives were acting in your best interest?  never (1)  rarely (2)  some of the time (3)  a good bit of the time (4)  usually (5)  always (6)  *Total* | 0 (0.00)  0 (0.00)  0 (0.00)  0 (0.00)  9 (10.34)  78 (89.66)  87 (100.00) | 1 (1.32)  1 (1.32)  8 (10.53)  12 (15.79)  15 (21.05)  38 (50.00)  76 (100.00) | 1 (0.61)  1 (0.61)  8 (4.91)  12 (7.36)  24 (15.34)  116 (71.17)  163 (100.00) |
| How often did you trust what your midwives told you?  never (1)  rarely (2)  some of the time (3)  a good bit of the time (4)  usually (5)  always (6)  *Total* | 0 (0.00)  0 (0.00)  1 (1.15)  2 (2.30)  16 (18.39)  68 (78.16)  87 (100.00) | 1 (1.32)  2 (2.63)  9 (11.84)  9 (11.84)  23 (31.58)  32 (40.79)  76 (100.00) | 1 (0.61)  2 (1.23)  10 (6.13)  11 (6.75)  39 (24.54)  100 (60.74)  163 (100.00) |
| How often did your midwives do what they said they would do?  never (1)  rarely (2)  some of the time (3)  a good bit of the time (4)  usually (5)  always (6)  *Total* | 0 (0.00)  0 (0.00)  1 (1.15)  1 (1.15)  18 (20.69)  67 (77.01)  87 (100.00) | 1 (1.30)  3 (3.90)  7 (9.09)  8 (10.39)  23 (31.17)  34 (44.16)  76 (100.00) | 1 (0.61)  3 (1.83)  8 (4.88)  9 (5.49)  41 (25.61)  101 (61.59)  163 (100.00) |
| How often did your midwives provide accurate information about the pregnancy?  never (1)  rarely (2)  some of the time (3)  a good bit of the time (4)  usually (5)  always (6)  *Total* | 0 (0.00)  0 (0.00)  0 (0.00)  3 (3.45)  8 (9.20)  76 (87.36)  87 (100.00) | 1 (1.30)  2 (2.60)  7 (9.09)  14 (18.18)  19 (24.68)  33 (44.16)  76 (100.00) | 1 (0.61)  2 (1.22)  7 (4.27)  17 (10.37)  27 (16.46)  109 (67.07)  163 (100.00) |
|  | POPPIE Mean  [95% CI] | Standard Mean  [95% CI] | Mean Diff [95% CI] |
| **TNS Score** (sum of response scores) | 28.89  [28.47, 29.31] | 24.68  [23.43, 25.92] | -4.21  [-5.44, -2.97] |
| (z= -6.438 p<0.0001) | | | |
| **Global item**: please rate how much you trusted your midwives on a scale from 1 to 10, with 1 indicating that you did not trust your nurses at all and 10 indicating that you trusted your nurses as much as possible: | 9.55  [9.38, 9.71] | 8.14  [7.73, 8.56] | -1.40 (0.21)  [-1.83, -0.98] |
| (z= -5.937, p<0.0001) | | | |

| **Perceptions of safety (AN) Please rate each of the following items by inserting a number between 1 and 5 in each box in order of importance in making you feel safe during your antenatal care (*1 less important; 5 most important)*** | **POPPIE**  **n (%)** | **Standard**  **n (%)** | *Total* |
| --- | --- | --- | --- |
| I could have a member of my family or close friend for support when I wanted:  1 (less important)  2  3  4  5 (more important)  *Total* | 12 (13.33)  7 (7.78)  13 (14.44)  10 (11.11)  48 (53.33)  90 (100.00) | 7 (9.21)  3 (3.95)  5 (6.58)  13 (17.11)  48 (63.16)  76 (100.00) | 19 (11.45)  10 (6.02)  18 (10.84)  23 (13.86)  96 (57.83)  166 (100.00) |
| My consent was obtained before a test, an investigation or an emergency procedure  1 (less important)  2  3  4  5 (more important)  *Total* | 5 (5.56)  4 (4.44)  10 (11.11)  13 (14.44)  58 (64.44)  90 (100.00) | 4 (5.26)  7 (9.21)  8 (10.53)  15 (19.74)  42 (55.26)  76 (100.00) | 9 (5.42)  11 (6.63)  18 (10.84)  28 (16.87)  100 (60.24)  166 (100.00) |
| Test and procedures were carried out when staff said they would be  1 (less important)  2  3  4  5 (more important)  *Total* | 0 (0.00)  3 (3.33)  14 (15.56)  18 (20.00)  55 (61.11)  90 (100.00) | 7 (9.21)  5 (6.58)  14 (18.42)  18 (23.68)  32 (42.11)  76 (100.00) | 7 (4.22)  8 (4.82)  28 (16.87)  36 (21.69)  87 (52.41)  166 (100.00) |
| There were always enough staff to care  1 (less important)  2  3  4  5 (more important)  Total | 3 (3.33)  3 (3.33)  5 (5.56)  21 (23.33)  58 (64.44)  90 (100.00) | 5 (6.58)  7 (9.21)  19 (25.00)  17 (22.37)  28 (36.84)  76 (100.00) | 8 (4.82)  10 (6.02)  24 (14.46)  38 (22.89)  86 (51.81)  166 (100.00) |
| Staff were familiar with the equipment and procedures  1 (less important)  2  3  4  5 (more important)  *Total* | 4 (4.44)  6 (6.67)  5 (5.56)  10 (11.11)  65 (72.22)  90 (100.00) | 7 (9.21)  4 (5.26)  10 (13.16)  13 (17.11)  42 (55.26)  76 (100.00) | 11 (6.63)  10 (6.02)  15 (9.04)  23 (13.86)  107 (64.46)  166 (100.00) |
| If applicable, I was given information about my medication in a way I could understand  1 (less important)  2  3  4  5 (more important)  *Total* | 2 (2.56)  1 (1.28)  5 (6.41)  11 (14.10)  59 (75.64)  78 (100.00 | 5 (8.06)  3 (4.84)  7 (11.29)  11 (17.74)  36 (58.06)  62 (100.00) | 7 (5.00)  4 (2.86)  12 (8.57)  22 (15.71)  95 (67.86)  140 (100.00) |
|  | POPPIE Mean  [95% CI] | Standard Mean  [95% CI] | Mean Diff [95% CI] |
| **AN Perceptions of Safety Score** (sum of response scores) | 25.31  (24.27, 26.32) | 23.28 (22.03, 24.54) | -2.01  (-3.01, 0.47) |
| (t=-2.4903; df=163; p= 0.0138) |  |  |  |
|  | | | |

| **Perceptions of safety intrapartum (IP). Please rate each of the following items by inserting a number between 1 and 5 in each box in order of importance in making you feel safe during your intrapartum care (1 less important; 5 most important)** | **POPPIE**  **n (%)** | **Standard**  **n (%)** | *Total* |
| --- | --- | --- | --- |
| I could have a member of my family or close friend for support when I wanted:  1 (less important)  2  3  4  5 (more important)  *Total* | 10 (11.11)  2 (2.22)  8 (8.89)  7 (7.78)  63 (70.00)  90 (100.00) | 7 (9.21)  0 (0.00)  3 (3.95)  4 (5.26)  62 (81.58)  76 (100.00) | 17 (10.24)  2 (1.20)  11 (6.63)  11 (6.63)  125 (75.30)  166 (100.00) |
| My consent was obtained before a test, an investigation or an emergency procedure  1 (less important)  2  3  4  5 (more important)  *Total* | 5 (5.56)  3 (3.33)  9 (10.00)  15 (16.67)  58 (64.44)  90 (100.00) | 0 (0.00)  8 (10.53)  8 (10.53)  14 (18.42)  46 (60.53)  76 (100.00) | 5 (3.01)  11 (6.63)  17 (10.24)  29 (17.47)  104 (62.65)  166 (100.00) |
| Test and procedures were carried out when staff said they would be  1 (less important)  2  3  4  5 (more important)  Total | 2 (2.22)  6 (6.67)  10 (11.11)  18 (20.00)  54 (60.00)  90 (100.00) | 6 (7.89)  5 (6.58)  18 (23.68)  12 (15.79)  35 (46.05)  76 (100.00) | 8 (4.82)  11 (6.63)  28 (16.87)  30 (18.07)  89 (53.61)  166 (100.00) |
| There were always enough staff to care  1 (less important)  2  3  4  5 (more important)  Totals | 4 (4.44)  3 (3.33)  8 (8.89)  16 (17.78)  59 (65.56)  90 (100.00) | 8 (10.53)  7 (9.21)  5 (6.58)  15 (19.74)  41 (53.95)  76 (100.00) | 12 (7.23)  10 (6.02)  13 (7.83)  31 (18.67)  100 (60.24)  166 (100.00) |
| Staff were familiar with the equipment and procedures  1 (less important)  2  3  4  5 (more important)  Total | 4 (4.44)  6 (6.67)  6 (6.67)  15 (16.67)  59 (65.56)  90 (100.00) | 5 (6.58)  2 (2.63)  8 (10.53)  11 (14.47)  50 (65.79)  76 (100.00) | 9 (5.42)  8 (4.82)  14 (8.43)  26 (15.66)  109 (65.66)  166 (100.00) |
| If applicable, I was given information about my medication in a way I could understand  1 (less important)  2  3  4  5 (more important)  Total | 3 (4.00)  0 (0.00)  1 (1.33)  14 (18.67)  57 (76.00)  75 (100.00 | 3 (4.76)  2 (3.17)  8 (12.70)  12 (19.05)  38 (60.32)  63 (100.00) | 6 (4.35)  2 (1.45)  9 (6.52)  26 (18.84)  95 (68.84)  140 (100.00) |
| **IP Perceptions of Safety Score** (sum of response scores) | 25.37  [24.24,26.51] | 24.46  [23.15, 25.76] | - 0.91  [-2.62, 0.78] |
| (t=-1.0611; df=163; p= 0.2902) |  |  |  |
|  | | | |

| **Perceptions of safety PN. Please rate each of the following items by inserting a number between 1 and 5 in each box in order of importance in making you feel safe during your postnatal care (1 less important; 5 most important)** | **POPPIE**  **n (%)** | **Standard**  **n (%)** | *Total* |
| --- | --- | --- | --- |
| I could have a member of my family or close friend for support when I wanted:  1 (less important)  2  3  4  5 (more important)  *Total* | 7 (8.05)  6 (6.90)  9 (10.34)  14 (16.19)  51 (58.62)  87 (100.00) | 5 (6.58)  3 (3.95)  11 (14.47)  2 (2.63)  55 (72.37)  76 (100.00) | 12 (7.36)  9 (5.52)  20 (12.27)  16 (9.82)  106 (65.03)  163 (100.00) |
| My consent was obtained before a test, an investigation or an emergency procedure  1 (less important)  2  3  4  5 (more important)  *Total* | 4 (4.60)  5 (5.75)  9 (10.34)  14 (16.09)  55 (63.22)  87 (100.00) | 5 (6.58)  6 (7.89)  8 (10.53)  15 (19.74)  42 (55.26)  76 (100.00) | 9 (5.52)  11 (6.75)  17 (10.43)  29 (17.79)  97 (59.51)  163 (100.00) |
| Test and procedures were carried out when staff said they would be  1 (less important)  2  3  4  5 (more important)  *Total* | 1 (1.15)  6 (6.90)  11 (12.64)  20 (22.99)  49 (56.32)  87 (100.00) | 6 (7.89)  7 (9.21)  12 (15.79)  12 (15.79)  39 (51.32)  76 (100.00) | 7 (4.29)  13 (7.98)  23 (14.11)  32 (19.63)  88 (53.99)  163 (100.00) |
| There were always enough staff to care  1 (less important)  2  3  4  5 (more important)  *Total* | 2 (2.30)  3 (3.45)  11 (12.64)  18 (20.69)  53 (60.92)  87 (100.00) | 3 (3.95)  4 (5.26)  13 (17.11)  16 (21.05)  40 (52.63)  76 (100.00) | 5 (3.07)  7 (4.29)  24 (14.72)  34 (20.86)  93 (57.06)  163 (100.00) |
| Staff were familiar with the equipment and procedures  1 (less important)  2  3  4  5 (more important)  Total | 2 (2.30)  4 (4.60)  9 (10.34)  13 (14.94)  59 (67.82)  87 (100.00) | 8 (10.53)  4 (5.26)  8 (10.53)  11 (14.47)  45 (59.21)  76 (100.00) | 10 (6.13)  8 (4.91)  17 (10.43)  24 (14.72)  104 (63.80)  163 (100.00) |
| If applicable, I was given information about my medication in a way I could understand  1 (less important)  2  3  4  5 (more important)  Total | 3 (3.90)  2 (2.60)  9 (11.69)  9 (11.69)  54 (70.13)  77 (100.00 | 3 (4.62)  2 (3.08)  10 (15.38)  12 (18.46)  38 (58.46)  65 (100.00) | 6 (4.23)  4 (2.82)  19 (13.38)  21 (14.79)  92 (64.79)  142 (100.00) |
| My discharge and my babies were well planned  1 (less important)  2  3  4  5 (more important)  Total | 6 (7.41)  0 (0.00)  11 (13.58)  15 (18.52)  49 (60.49)  81 (100.00) | 6 (8.57)  4 (5.71)  13 (18.57)  12 (17.14)  35 (50.00)  70 (100.00) | 12 (7.95)  4 (2.65)  24 (15.89)  27 (17.88)  84 (55.63)  151 (100.00) |
|  | POPPIE Mean  [95% CI] | Standard Mean  [95% CI] | Mean Diff [95% CI] |
| **PN Perceptions of Safety Score** (sum of response scores) | 28.28 [26.69,29.88] | 27.78  [28.09, 29.25] | -0.51  [-2.69, 1.67] |
| (t= -0.4635; df=163; p= 0.6436) |  |  |  |
|  | | | |

| **Labour Agentry Scale (LAS). Please try to recall your labour and your baby’s birth as vividly as you can. Think about your feelings during labour and birth. Of course, you probably had many different feelings, but try to remember what it was generally like for you during this time.** | **POPPIE**  **n (%)** | **Standard**  **n (%)** | *Total* |  |
| --- | --- | --- | --- | --- |
| 1. I felt tense  almost all of the time (1)  a lot but not always (2)  a little more than half the time (3)  about half the time (4)  slightly less than half the time (5)  sometimes (6)  never or almost never (7)  *Total* | 11 (12.22)  12 (13.13)  7 (7.78)  7 (7.78)  6 (6.67)  30 (33.33)  17 (18.89)  90 (100.00) | 10 (13.16)  12 (15.79)  8 (10.53)  10 (13.16)  1 (1.32)  21 (27.63)  14 (18.42)  76 (100.00) | 21 (12.65)  24 (14.46)  15 (9.04)  17 (10.24)  7 (4.22)  51 (30.72)  31 (18.67)  166 (100.00) |  |
| 2. I felt important  almost all of the time (7)  a lot but not always (6)  a little more than half the time (5)  about half the time (4)  slightly less than half the time (3)  sometimes (2)  never or almost never (1)  *Total* | 54 (60.00)  13 (14.44)  7 (7.78)  5 (5.56)  3 (3.33)  6 (6.67)  2 (2.22)  90 (100.00) | 35 (46.05)  14 (18.42)  7 (9.21)  6 (7.89)  2 (2.63)  7 (9.21)  5 (6.58)  76 (100.00) | 89 (53.61)  27 (16.27)  14 (8.43)  11 (6.63)  5 (3.01)  13 (7.83)  7 (4.22)  166 (100.00) |  |
| 3. I felt confident  almost all of the time (7)  a lot but not always (6)  a little more than half the time (5)  about half the time (4)  slightly less than half the time (3)  sometimes (2)  never or almost never (1)  *Total* | 21 (23.34)  25 (27.78)  13 (14.44)  11 (12.22)  6 (6.67)  9 (10.00)  5 (5.56)  90 (100.00) | 15 (28.95)  22 (28.95)  6 (7.89)  7 (9.21)  7 (9.21)  15 (19.74)  4 (5.26)  76 (100.00) | 36 (21.69)  47 (28.31)  19 (11.45)  18 (10.84)  13 (7.83)  24 (14.46)  9 (5.42)  166 (100.00) |  |
| 4. I felt in control  almost all of the time (7)  a lot but not always (6)  a little more than half the time (5)  about half the time (4)  slightly less than half the time (3)  sometimes (2)  never or almost never (1)  *Total* | 20 (22.23)  24 (26.67)  14 (15.56)  8 (8.89)  4 (4.44)  11 (12.22)  9 (10.00)  90 (100.00) | 14 (18.42)  19 (25.00)  7 (9.21)  11 (14.47)  8 (10.53)  10 (13.16)  7 (9.21)  76 (100.00) | 34 (20.48)  43 (25.90)  21 (12.65)  19 (11.45)  12 (7.23)  21 (12.65)  16 (9.64)  166 (100.00) |  |
| 5. I felt fearful  almost all of the time (1)  a lot but not always (2)  a little more than half the time (3)  about half the time (4)  slightly less than half the time (5)  sometimes (6)  never or almost never (7)  *Total* | 9 (10.00)  10 (11.11)  12 (13.33)  6 (6.67)  7 (7.78)  22 (24.44)  24 (26.67)  90 (100.00) | 9 (11.84)  7 (9.21)  8 (10.53)  9 (11.84)  5 (6.58)  22 (28.95)  16 (21.05)  76 (100.00) | 7 (4.22)  3 (7.83)  5 (3.01)  11 (6.63)  14 (8.43)  27 (16.27)  89 (53.61)  166 (100.00) |  |
| 6. I felt relaxed  almost all of the time (7)  a lot but not always (6)  a little more than half the time (5)  about half the time (4)  slightly less than half the time (3)  sometimes (2)  never or almost never (1)  *Total* | 15 (16.67)  12 (13.33)  16 (17.78)  9 (10.00)  6 (6.67)  20 (22.22)  12 (13.33)  90 (100.00) | 11 (14.47)  11 (14.47)  5 (6.58)  13 (17.11)  6 (7.89)  11 (14.47)  19 (25.00)  76 (100.00) | 31 (18.67)  31 (18.67)  12 (7.23)  22 (13.25)  21 (12.65)  23 (13.86)  26 (15.66)  166 (100.00) |  |
| 7. I felt good about my behavior  almost all of the time (7)  a lot but not always (6)  a little more than half the time (5)  about half the time (4)  slightly less than half the time (3)  sometimes (2)  never or almost never (1)  *Total* | 35 (38.89)  19 (21.11)  9 (10.00)  9 (10.00)  6 (6.67)  10 (11.11)  2 (2.22)  90 (100.00) | 32 (42.11)  24 (31.58)  5 (6.58)  7 (9.21)  0 (0.00)  5 (6.58)  3 (3.95)  76 (100.00) | 67 (40.36)  43 (25.90)  14 (8.43)  16 (9.64)  6 (3.61)  5 (3.01)  15 (9.04)  166 (100.00) |  |
| 8. I felt helpless (powerless)  almost all of the time (1)  a lot but not always (2)  a little more than half the time (3)  about half the time (4)  slightly less than half the time (5)  sometimes (6)  never or almost never (7)  *Total* | 10 (11.11)  6 (6.67)  5 (5.56)  5 (5.56)  5 (5.56)  21 (23.33)  38 (42.22)  90 (100.00) | 4 (5.26)  10 (13.16)  3 (3.95)  7 (9.21)  1 (1.32)  16 (21.05)  35 (46.05)  76 (100.00) | 14 (8.43)  16 (9.64)  8 (4.82)  12 (7.23)  6 (3.61)  37 (22.29)  73 (43.98)  166 (100.00) |  |
| 9. I felt I was with people who care about me  almost all of the time (7)  a lot but not always (6)  a little more than half the time (5)  about half the time (4)  slightly less than half the time (3)  sometimes (2)  never or almost never (1)  *Total* | 63 (70.00)  15 (16.67)  4 (4.44)  4 (4.44)  3 (3.33)  1 (1.11)  0 (0.00)  90 (100.00) | 50 (65.79)  10 (13.16)  5 (6.58)  3 (3.95)  1 (1.32)  5 (6.58)  2 (2.63)  76 (100.00) | 113 (68.07)  25 (15.06)  9 (5.42)  7 (4.22)  4 (2.41)  6 (3.61)  2 (1.20)  166 (100.00) |  |
| 10. I felt like a failure  almost all of the time (1)  a lot but not always (2)  a little more than half the time (3)  about half the time (4)  slightly less than half the time (5)  sometimes (6)  never or almost never (7)  *Total* | 3 (3.33)  2 (2.22)  5 (5.56)  7 (7.78)  3 (3.33)  8 (8.89)  62 (68.89)  90 (100.00) | 2 (2.63)  2 2.63)  2 (2.63)  1 (1.32)  0 (0.00)  9 (11.84)  60 (78.95)  76 (100.00) | 5 (3.01)  4 (2.41)  7 (4.22)  8 (4.82)  3 (1.81)  17 (10.24)  122 (73.5)  166 (100.00) |  |
|  | Mean  [95% CI] | Mean  [95% CI] | Mean Diff  [95% CI] |  |
| **LAS score** | 52.12  [49.41 to 54.82] | 50.69  [47.78 to 53.61] | -1.42  [-5.37 to 2.52] | |
| (t= -0.7120, df=163, p=0.4775) | | | |  |

| **Mother-to-Infant Bonding Scale (MIBS) The following statements are about your feelings for your child in the first few weeks. Some adjectives are listed below which describe some feelings mothers have towards their baby after they are born. Please make a tick again each word in the box that described how you felt during the FIRST FEW WEEKS:** | **POPPIE**  **n (%)** | **Standard**  **n (%)** | *Total* |
| --- | --- | --- | --- |
| Loving  very much (0)  a lot (1)  a little (2)  not at all (3)  *Total* | 73 (83.91)  10 (11.45)  4 (4.60)  0 (0.00)  87 (100.00) | 63 (82.89)  9 (11.84)  2 (2.63)  2 (2.63)  76 (100.00) | 136 (83.44)  19 (11.66)  6 (3.68)  2 (1.23)  163 (100.00) |
| Resentful  very much (3)  a lot (2)  a little (1)  not at all (o)  *Total* | 3 (3.45)  3 (3.45)  7 (8.05)  74 (85.06)  87 (100.00) | 2 (2.63)  3 (3.95)  12 (15.79)  59 (77.63)  76 (100.00) | 5 (3.07)  6 (3.68)  19 (11.66)  133 (81.60)  163 (100.00) |
| Neutral or felt nothing  very much (3)  a lot (2)  a little (1)  not at all (o)  *Total* | 1 (1.15)  1 (1.15)  11 (12.64)  74 (85.06)  87 (100.00) | 1 (1.32)  2 (2.63)  11 (14.47)  62 (81.58)  76 (100.00) | 2 (1.23)  3 (1.84)  22 (13.50)  136 (83.44)  163 (100.00) |
| joyful  very much (3)  a lot (2)  a little (1)  not at all (o)  *Total* | 62 (81.58)  16 (18.39)  7 (8.05)  2 (2.30)  87 (100.00) | 40 (52.63)  25 (32.89)  7 (9.21)  4 (5.26)  76 (100.00) | 102 (62.58)  41 (25.15)  14 (8.59)  6 (3.68)  163 (100.00) |
| Dislike  very much  a lot  a little  not at all  *Total* | 0 (0.00)  1 (1.15)  5 (5.75)  85 (93.10)  87 (100.00) | 0 (0.00)  0 (0.00)  5 (6.58)  71 (93.42)  76 (100.00) | 0 (0.00)  1 (0.61)  10 (6.13)  153 (93.25)  163 (100.00) |
| Protective  very much (0)  a lot (1)  a little (2)  not at all (3)  *Total* | 69 (79.31)  14 (16.09)  3 (3.45)  1 (1.15)  87 (100.00) | 67 (88.31)  4 (5.19)  4 (5.19)  1 (1.30)  76 (100.00) | 136 (83.54)  18 (10.98)  7 (4.27)  2 (1.22)  163 (100.00) |
| Disappointed  very much (3)  a lot (2)  a little (1)  not at all (o)  *Total* | 0 (0.00)  0 (0.00)  10 (11.49)  77 (88.51)  87 (100.00) | 1 (1.32)  1 (1.32)  10 (13.16)  64 (84.21)  76 (100.00) | 1 (0.61)  1 (0.61)  20 (17.27)  141 (86.50)  163 (100.00) |
| Aggressive  very much (3)  a lot (2)  a little (1)  not at all (o)  *Total* | 0 (0.00)  0 (0.00)  2 (2.30)  85 (97.70)  87 (100.00) | 0 (0.00)  0 (0.00)  5 (6.58)  71 (93.42)  76 (100.00) | 0 (0.00)  0 (0.00)  7 (4.29)  156 (95.71)  163 (100.00) |
|  | Mean  [95% CI] | Mean  [95% CI] | Mean Diff  [95% CI] |
| **MBIS score** | 1.54  [1.07 to 2.01] | 1.97 (2.63)  [1.37 to 2.57] | 0.45  [-0.28, 1.20] |
| (z= 1.258, p= 0.2085) | | | |
| \| **PROMIS-10. In the following questions, please tick the answer which comes closest to your health and your quality of life:** \| **POPPIE**  **n (%)** \| **Standard**  **n (%)** \| *Total* \| \| --- \| --- \| --- \| --- \| \| 1.In the past few weeks…In general, would you say your health is  Excellent (5)  Very good (4)  Good (3)  Fair (2)  Poor (1)  *Total* \| 18 (20.69)  32 (36.78)  29 (33.33)  8 (9.20)  0 (0.00)  87 (100.00) \| 21 (27.63)  31 (40.79)  18 (23.68)  5 (6.58)  1 (1.32)  76 (100.00) \| 39 (23.93)  63 (38.65)  47 (28.83)  13 (7.98)  1 (0.61)  163 (100.00) \| \| 2.In the past few weeks…In general, would you say your quality of life  Excellent (5)  Very good (4)  Good (3)  Fair (2)  Poor (1)  *Total* \| 19 (21.84)  39 (44.83)  20 (20.99)  8 (9.20)  1 (1.15)  87 (100.00) \| 22 (28.95)  30 (39.47)  19 (25.00)  5 (6.58)  0 (0.00)  76 (100.00) \| 41 (25.15)  69 (42.33)  39 (23.93)  13 (7.98)  1 (0.61)  163 (100.00) \| \| 3.In the past few weeks…In general, how would you rate your physical health  Excellent (5)  Very good (4)  Good (3)  Fair (2)  Poor (1)  *Total* \| 15 (17.24)  29 (18.39)  25 (28.74)  16 (18.39)  2 (2.30)  87 (100.00) \| 14 (18.42)  25 (32.89)  29 (38.16)  7 (9.21)  1 (1.32)  76 (100.00) \| 29 (17.79)  54 (33.12)  54 (33.12)  23 (14.11)  3 (1.84)  163 (100.00) \| \| 4.In the past few weeks… how would you rate your mental health, including your mood and your ability to think  Excellent (5)  Very good (4)  Good (3)  Fair (2)  Poor (1)  *Total* \| 22 (25.29)  30 (34.48)  24 (27.59)  9 (10.39)  2 (2.30)  87 (100.00) \| 19 (25.00)  28 (36.84)  21 (27.63)  5 (6.58)  3 (3.95)  76 (100.00) \| 41 (25.15)  58 (35.58)  45 (27.61)  14 (8.59)  5 (3.07)  163 (100.00) \| \| 5.In the past few weeks…In general, how would you rate your satisfaction with your social activities and relationships  Excellent (5)  Very good (4)  Good (3)  Fair (2)  Poor (1)  *Total* \| 19 (21.84)  28 (32.18)  25 (28.74)  11 (12.64)  4 (4.60)  87 (100.00) \| 15 (19.74)  25 (34.21)  21 (27.63)  12 (15.79)  4 (2.63)  76 (100.00) \| 34 (20.86)  54 (33.13)  46 (28.22)  23 (14.11)  6 (3.68)  163 (100.00) \| \| 6.In the past few weeks…In general, please rate how well you carry out your usual social activities and roles. This includes activities at home, at work and in the community, and responsibilities as a parent, spouse, employee, friend, etc  Excellent (5)  Very good (4)  Good (3)  Fai  Poor (1)  *Total* \| 21 (24.14)  27 (31.03)  22 (25.29)  12 (13.79)  5 (5.75)  87 (100.00) \| 14 (18.42)  20 (26.32)  28 (36.84)  8 (10.53)  6 (7.89)  76 (100.00) \| 35 (21.47)  47 (28.83)  50 (30.67)  20 (12.27)  11 (6.75)  163 (100.00) \| \| 7.In the past few weeks…To what extent are you able to carry out your everyday physical activities such as walking, climbing stairs, etc  Completely (5)  Mostly (4)  Moderately (3)  A little (2)  Not a lot (1)  *Total* \| 48 (55.17)  25 (28.74)  10 (11.49)  3 (3.45)  1 (1.15)  87 (100.00) \| 41 (53.95)  24 (31.58)  9 (11.84)  1 (1.32)  1 (1.32)  76 (100.00) \| 89 (54.60)  49 (30.06)  19 (11.66)  4 (2.45)  2 (1.23)  163 (100.00) \| \| 8. In the past days... How often have you been bothered by emotional problems such as feeling an anxious, depressed or irritable?  Never (5)  Rarely (4)  Sometimes (3)  Often (2)  Always (1)  *Total* \| 27 (31.03)  24 (27.59)  17 (19.54)  16 (18.39)  3 (3.45)  87 (100.00) \| 21 (27.63)  21 (27.63)  19 (25.00)  13 (17.11)  2 (2.63)  76 (100.00) \| 48 (29.45)  45 (27.61)  36 (22.09)  29 (17.79)  5 (3.07)  163 (100.00) \| \| **9**.In the past 7 days… How would you rate your fatigue on average?  None (5)  Mild (4)  Moderate (3)  Severe (2)  Very severe (1)  *Total* \| 12 (13.79)  63 (72.41)  11 (12.64)  0 (0.00()  1 (1.15)  87 (100.00) \| 10 (13.16)  59 (77.63)  6 (7.89)  0 (0.00)  1 (1.32)  76 (100.00) \| 22 (13.50)  122 (74.85)  17 (10.43)  0 (0.00)  2 (1.23)  163 (100.00) \| \| 10.How would you rate your pain on average?  0 (5)  1,2,3 (4)  4,5,6 (3)  7,8,9 (2)  10 (1)  *Total* \| 12 (13.79)  23 (26.44)  40 (45.98)  11 (12.64)  1 (1.15)  87 (100.00) \| 10 (13.16)  22 (28.95)  37 (48.68)  6 (7.89)  1 (1.32)  76 (100.00) \| 22 (13.50)  45 (27.61)  77 (47.24)  17 (10.43)  2 (1.23)  163 (100.00) \| \|  \| Mean (SD)  [95% CI] \| Mean (SD)  [95% CI] \| Mean Diff (SD) [95% CI] \| \| GPH scores:  Raw score  T-score \| 15.43 (2.73)  [14.85, 16.02]  47.7 (4.4) \| 15.80 (2.29)  [15.27, 16.32]  47.7 (4.4) \| 0.36  (-0.42, 1.15) \| \| t=0.9162; df=161; p=0.3610 \| \| \| \| \| GMH scores:  Raw scores    T-Score \| 14.65 (3.67)  [13.87, 15.43]  48.3 (3.7) \| 14.76 (3.37)  [13.99, 15.53]  48.3 (3.7) \| 0.11  (-0.98, 1.20) \| \| t=0.1944; df=161; p=0.8461 \| \| \| \|   **4. Details of additional questions: numbers, proportions and statistical tests.**   \| **Were you able to contact your midwife when you needed in the antenatal period?** \| **POPPIE**  **n (%)** \| **Standard**  **n (%)** \| *Total* \| \| --- \| --- \| --- \| --- \| \| No \| 1 (1.11) \| 25 (32.89) \| 26 (15.66) \| \| Yes \| 89 (98.89) \| 51 (67.11) \| 140 (84.34) \| \| *Total* \| 90 (100.00) \| 76 (100.00) \| 166 (100.00) \| \| (χ^2^ = 31.5115, df=1, p<0.0001) \| \| \| \|  \| **If yes, how did you contact her?** \| **POPPIE**  **n (%)** \| **Standard**  **n (%)** \| *Total* \| \| --- \| --- \| --- \| --- \| \| Mobile phone (i.e. calls, texts) \| 85 (95.5) \| 38 (74.5) \| 123 (87.8) \| \| Mobile phone (i.e. calls, texts) and email \| 1 (1.12) \| 1 (1.96) \| 2 (1.42) \| \| Other phones (i.e. labour, community, DAU) \| 0 (0.0) \| 3 (5.88) \| 3 (2.14) \| \| Blank / Not documented \| 3 (3.37) \| 9 (17.6) \| 12 (8.57) \| \| *Total* \| 89 (100.00) \| 51 (100.00) \| 140 (100.00) \| \|  \| \| \| \|  \| **Were you able to contact your midwife when you needed in the postnatal period?** \| **POPPIE**  **n (%)** \| **Standard**  **n (%)** \| *Total* \| \| --- \| --- \| --- \| --- \| \| No \| 0 (0.00) \| 29 (38.16) \| 29 (17.68) \| \| Yes \| 88 (100.00) \| 47 (61.84) \| 135 (82.32) \| \| *Total* \| 88 (100.00) \| 76 (100) \| 164 (100.00) \| \| (χ^2^ test; p<0.0001= 40.7922, df=1, p<0.0001) \| \| \| \|  \| **If yes, how did you contact her?** \| **POPPIE**  **n (%)** \| **Standard**  **n (%)** \| *Total* \| \| --- \| --- \| --- \| --- \| \| Mobile phone (i.e. calls, texts) \| 86 (97.72) \| 32 (69.08) \| 118 (87.40) \| \| Other phones (i.e. labour ward, community, DAU) \| 0 (0.0) \| 4 (8.51) \| 4 (2.96) \| \| Helpline \| 0 (0.0) \| 1 (2.12) \| 1 (0.74) \| \| Blank / Not documented \| 2 (2.27) \| 10 (21.27) \| 12 (8.88) \| \| *Total* \| 88 (100.00) \| 47 (100.00) \| 135 (100.00) \| \|  \| \| \| \|  \| **Thinking about your antenatal care, were you spoken to in a way you could understand?** \| **POPPIE**  **n (%)** \| **Standard**  **n (%)** \| *Total* \| \| --- \| --- \| --- \| --- \| \| Yes \| 90 (100) \| 74 (97.37) \| 164 (98.8) \| \| No \| 0 (0.00) \| 2 (2.63) \| 2 (1.20) \| \| Unsure \| 0 (0.00) \| 0 (0.00) \| 0 (0.00) \| \| *Total* \| 90 (100.00) \| 76 (100.00) \| 166 (100.00) \| \| (χ^2^ =16.9901, df=2, p<0.0001) \| \| \| \| | | | |

| **Would you prefer to have been more or less involved in the decisions about your antenatal care?** | **POPPIE**  **n (%)** | **Standard**  **n (%)** | *Total* |
| --- | --- | --- | --- |
| Less involved in the decision making | 0 (0.00) | 1 (1.32) | 1 (1.32) |
| More involved in the decision making | 5 (5.56) | 18 (23.68 | 23 (13.86) |
| I was happy with how involved I was | 85 (94.44) | 57 (75.00) | 142 (85.54) |
| *Total* | 90 (100.00) | 76 (100.00) | 166 (100.00) |
| (χ^2^ =12.7791, df=2, p= 0.002) | | | |

| **Would you prefer to have been more or less involved in the decisions about your intrapartum care?** | **POPPIE**  **n (%)** | **Standard**  **n (%)** | *Total* |
| --- | --- | --- | --- |
| Less involved in the decision making | 0 (0.00) | 1 (1.32) | 1 (1.32) |
| More involved in the decision making | 12 (13.33) | 24 (31.58) | 36 (21.69) |
| I was happy with how involved I was | 78 (86.67) | 51 (67.11) | 129 (77.71) |
| *Total* | 90 (100.00) | 76 (100.00) | 166 (100.00) |
| (χ^2^ =9.5383, df=2, p=0.008) | | | |

| **Would you prefer to have been more or less involved in the decisions about your intrapartum care?** | **POPPIE**  **n (%)** | **Standard**  **n (%)** | *Total* |
| --- | --- | --- | --- |
| Less involved in the decision making | 0 (0.00) | 1 (1.32) | 1 (1.32) |
| More involved in the decision making | 7 (8.05) | 19 (25.00) | 26 (15.95) |
| I was happy with how involved I was | 80 (91.95) | 56 (73.68) | 136 (83.44) |
| *Total* | 87 (100.00) | 76 (100.00) | 163 (100.00) |
| (χ^2^ =10.0773, df=2, p= 0.006) | | | |

| **If you raised a concern during labour and birth, did you feel that it was taken seriously?** | **POPPIE**  **n (%)** | **Standard**  **n (%)** | *Total* |
| --- | --- | --- | --- |
| Yes | 67 (74.45) | 45 (59.22) | 112 (67.47) |
| No | 5 (5.56) | 11 (14.47) | 16 (9.64) |
| Not applicable | 18 (20.00) | 20 (26.32) | 38 (22.89) |
| *Total* | 90 (100.00) | 76 (100.00) | 166 (100.00) |
| (χ^2^ =5.5353, df=2, p=0.063) | | | |

| **Were you (and your companion if you had one) left alone during labour and birth by midwives or doctors at a time when it worried you?** | **POPPIE**  **n (%)** | **Standard**  **n (%)** | *Total* |
| --- | --- | --- | --- |
| Yes | 17 (22.37) | 12 (13.33) | 29 (17.47) |
| No | 69 (76.67) | 53 (69.74) | 122 (73.49) |
| Not applicable | 9 (10.00) | 6 (7.89) | 15 (9.04) |
| *Total* | 90 (100.00) | 76 (100.00) | 166 (100.00) |
| (χ^2^ = 2.3968, df=3, p= 0.302) | | | |

| **Were you given enough help and advice about each of the things listed below during your postnatal care?** | **POPPIE**  **n (%)** | **Standard**  **n (%)** | *Total* |
| --- | --- | --- | --- |
| Feeding the baby  Yes (always)  Yes (sometimes)  No  Unsure  *Total* | 71 (80.68)  14 (15.91)  3 (3.41)  0 (0.00)  88 (100.00) | 37 (48.68)  25 (32.89)  14 (3.41)  0 (0.00)  76 (100.00) | 108 (65.85)  39 (23.78)  17 (10.37)  0 (0.00)  164 (100.00) |
| (χ^2^ = 20.1538, df=2, p<0.0001) | | | |
| How to handle, settle and look after the baby  Yes (always)  Yes (sometimes)  No  Unsure  *Total* | 57 (64.77)  23 (26.14)  7 (7.95)  1 (1.14)  88 (100.00) | 27 (35.53)  22 (28.95)  25 (32.89)  2 (2.63)  76 (100.00) | 84 (51.22)  45 (27.44)  32 (19.51)  3 (1.83)  164 (100.00) |
| (χ^2^ = 20.4262, df=3, p<0.0001) | | | |
| Your baby’s health and progress and any problems  Yes (always)  Yes (sometimes)  No  Unsure  *Total* | 69 (78.41)  14 (15.91)  5 (5.68)  0 (0.00)  88 (100.00) | 46 (60.53)  24 (31.58)  6 (7.89)  0 (0.00)  76 (100.00) | 115 (70.12)  38 (23.17)  11 (6.71)  0 (0.00)  164 (100.00) |
| (χ^2^ =6.4791, df=2, p=0.039) | | | |
| Your own health and recovery after the birth  Yes (always)  Yes (sometimes)  No  Unsure  *Total* | 69 (78.41)  15 (17.05)  4 (4.55)  0 (0.00)  88 (100.00) | 42 (55.26)  28 (36.84)  6 (7.89)  0 (0.00)  76 (100.00) | 111 (67.68)  43 (26.22)  10 (6.10)  0 (0.00)  164 (100.00) |
| (χ^2^ =10.0737, df=2, p=0.006) | | | |
| Who to contact if you needed advice about any emotional changes  Yes (always)  Yes (sometimes)  No  Unsure  *Total* | 64 (72.73)  17 (19.32)  7 (7.95)  0 (0.0)  88 (100.00) | 36 (47.37)  25 (32.89)  12 (15.79)  3 (3.95)  76 (100.00) | 100 (60.98)  42 (25.61)  19 (11.59)  3 (3.95)  164 (100.00) |
| (χ^2^=12.8705, df=3, p=0.005) | | | |
|  | | | |
