# Supplementary material for: Experiences of maternity care among women at increased risk of preterm birth receiving midwifery continuity of care compared to women receiving standard care: Results from the POPPIE pilot trial
Source: PLoS One. 2021 Apr 21;16(4):e0248588. doi: 10.1371/journal.pone.0248588 (PMC8059847; doi:10.1371/journal.pone.0248588)
Supplement: S5 File — (DOCX) [file pone.0248588.s005.docx]

**S5 File: Interview Topic Guide for the POPPIE pilot trial**

**Interviews with women and partners:**

Journey through antenatal, intrapartum and postnatal care

Trust and continuity

Advocacy

Social capital / Social exclusion

Confidence

Engagement

Optimality

Wishes for future births

**Interviews with local stakeholders:**

Service configuration: consultations, reconfigurations and developments.

Service organisation: workforce arrangements, skill mix, models of care and escalation/transfer services and protocols

Expected outcomes and impact on organisation

Previous or current plans for change or development and reasons for these.

Implementation: context, barriers and facilitators

**Interviews with healthcare professionals:**

Experiences of working on or in relation to the POPPIE

Facilitators and barriers

The impact on service and women’s care

Training provision, support and development arrangements

Impact of their work and working patterns

The midwives’ work/life balance

Boundary working / Job satisfaction / jobs stress
